# Genomic characterization of a polyvalent hydrocarbonoclastic bacterium *Pseudomonas* sp. strain BUN14

**DOI:** 10.1038/s41598-021-87487-2

**Published:** 2021-04-14

**Authors:** Mouna Mahjoubi, Habibu Aliyu, Mohamed Neifar, Simone Cappello, Habib Chouchane, Yasmine Souissi, Ahmed Salaheddine Masmoudi, Don A. Cowan, Ameur Cherif

**Affiliations:** 1 University of Manouba, ISBST, BVBGR-LR11ES31, Biotechpole SidiThabet, 2020 Ariana, Tunisia; 2 Institute of Process Engineering in Life Science 2: Technical Biology, Karlsruhe Institute of Technology, Karlsruhe, Germany; 3 Istituto per le Risorse Biologiche e le Biotecnologie Marine (IRBIM)-CNR of Messina, Sp. San Raineri, 86, 98122 Messina, Italy; 4 Centre for Microbial Ecology and Genomics, University of Pretoria, Pretoria, 0002 South Africa

**Keywords:** Microbiology, Bacteria, Environmental microbiology

## Abstract

Bioremediation offers a viable alternative for the reduction of contaminants from the environment, particularly petroleum and its recalcitrant derivatives. In this study, the ability of a strain of *Pseudomonas* BUN14 to degrade crude oil, pristane and dioxin compounds, and to produce biosurfactants, was investigated. BUN14 is a halotolerant strain isolated from polluted sediment recovered from the refinery harbor on the Bizerte coast, north Tunisia and capable of producing surfactants. The strain BUN14 was assembled into 22 contigs of 4,898,053 bp with a mean GC content of 62.4%. Whole genome phylogeny and comparative genome analyses showed that strain BUN14 could be affiliated with two validly described *Pseudomonas* Type Strains, *P. kunmingensis* DSM 25974^T^ and *P. chloritidismutans* AW-1^T^. The current study, however, revealed that the two Type Strains are probably conspecific and, given the priority of the latter, we proposed that *P. kunmingensis* DSM 25974 is a heteronym of *P. chloritidismutans* AW-1^T^. Using GC-FID analysis, we determined that BUN14 was able to use a range of hydrocarbons (crude oil, pristane, dibenzofuran, dibenzothiophene, naphthalene) as a sole carbon source. Genome analysis of BUN14 revealed the presence of a large repertoire of proteins (154) related to xenobiotic biodegradation and metabolism. Thus, 44 proteins were linked to the pathways for complete degradation of benzoate and naphthalene. The annotation of conserved functional domains led to the detection of putative genes encoding enzymes of the rhamnolipid biosynthesis pathway. Overall, the polyvalent hydrocarbon degradation capacity of BUN14 makes it a promising candidate for application in the bioremediation of polluted saline environments.

## Introduction

Petroleum and dioxins compounds are omnipresent environmental pollutants that threaten the environment and human health due to their toxicological properties and their high resistance to degradation^1,2^. Bioremediation is considered as one of the viable options for remediation of hydrocarbon-polluted environments^3,4^. Numerous microorganisms, including bacteria, fungi, archaea and algae, have been investigated for their bioremediation potential^3,4^ with 79 bacterial genera reported to possess the capacity to degrade hydrocarbons^3^. Members of the Gammaproteobacteria class have high hydrocarbon-degrading capacity, and this taxon includes the most obligate hydrocarbonoclastic genera: *Alcanivorax, Thalassolituus*, *Oleiphilus, Oleispira, Cycloclasticus* and *Marinobacter*^3,5,6^. However, other non-obligate hydrocarbonoclastic members of the Gammaproteobacteria, such as the genus *Pseudomonas*, are well known for their hydrocarbon-degrading capacity^4,7^. The genetic basis and enzymatic mechanisms involved in the degradation of oil components, including alkanes and aromatic compounds, by *Pseudomonas* species have been reported in detail^8,9^. The key activating enzymes in the degradation of alkanes are alkane hydroxylases^10^. Alkane monooxygenases (AlkB) and cytochrome P450s (CYP153) catalyze the hydroxylation of alkanes to alcohols, which are subsequently oxidized to fatty acids. The fatty acid products are further metabolized by β-oxidation^10^. The catabolism of aromatic compounds involves multiple enzymes which convert the aromatic substrates into Krebs cycle intermediates via *ortho* or *meta*-cleavage pathways. The l3-ketoadipate pathway (*ortho-cleavage* pathway) is the key route for the catabolism of a wide variety of aromatic compounds through the protocatechuate branch (*pea* genes) and the catechol branch (*cat* genes).

The phylogeny of the genus *Pseudomonas* is complex, with some 219 validly published and correctly named species^11^. However, the current phylogeny is subject to considerable debate^12^ and many of the named species may be considered as conspecific. For example, *Pseudomonas kunmingensis* HL22-2T and *P. chloritidismutans* AW-1T which are closely related to *P. stutzeri* may not deserve separate species status^13,14^.

To date, the genome sequences of eighteen strains affiliated to *P. kunmingensis* and one affiliated to *P. chloritidismutans* strain are available via the NCBI database^15^. While the genes and pathways associated with n-alkane degradation have been reported for *P. chloritidismutans*^16,17^, similar information for *Pseudomonas kunmingensis* is not available.

*Pseudomonas* sp. BUN14, isolated from hydrocarbon-polluted sediments from the Bizerte coast refinery harbour, north Tunisia, exhibits the capacity for hydrocarbon degradation. In this study, we combined genomic analysis and degradation experiments to demonstrate that *Pseudomonas* strain BUN14 has potential for application in the bioremediation of hydrocarbon contaminated marine environments.

## Results and discussion

### Isolation, phylogenetic assignment, and characterization of hydrocarbonoclastic bacterial strain BUN14

Strain BUN14 was isolated on ONR7a mineral medium supplemented with 1% crude oil as the sole carbon source. The comparison of the strain BUN14 16S rRNA gene sequence with those of validly described strains in the EzBioCloud^18,19^ revealed that strain BUN14 showed highest similarity (99.23%) to that of *P. kunmingensis* HL22-2^T^ Phylogenetic analysis based on the 16S rRNA gene of *Pseudomonas* species placed *P. kunmingensis* HL22-2^T^ as the closest relative of BUN14 (Fig. S1). Growth of strain BUN14 on plates containing TSA (Trypticase soy agar) medium showed that the bacterium formed circular, opaque yellow colonies. Cells were rod-shaped and approximately 0.6 ± 0.1 μm in diameter and 1.8–2.0 μm in length (Fig. S2).

### Optimization of surfactant activities

**Figure 1:**
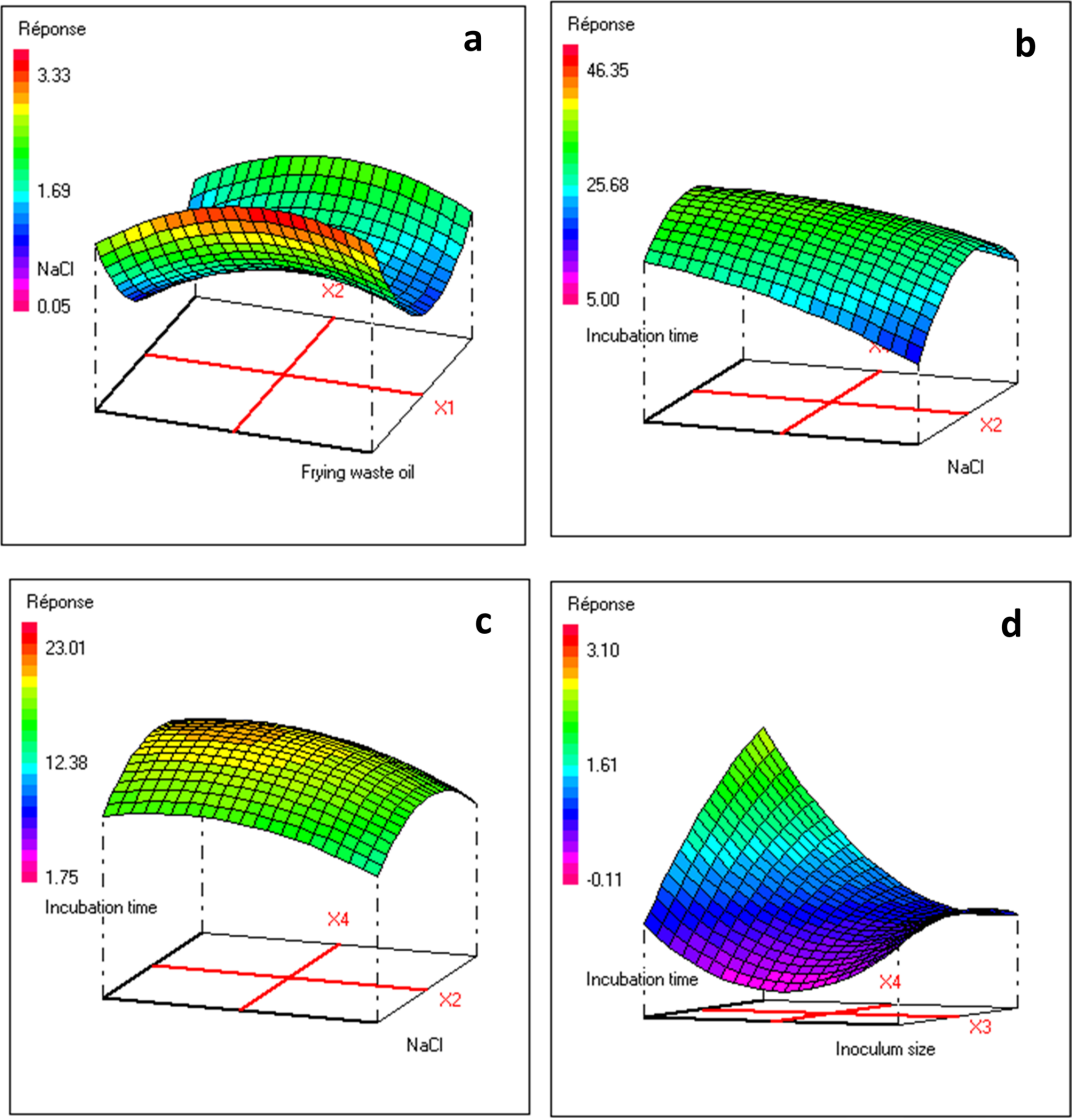
Three-dimensional response surface for the effect of waste frying oil, inoculum size, [NaCl] and incubation time on the response Y1 (**a**), Y2 (**b**), Y3 (**c**) and Y4 (**d**). The response surface graphs were generated by NEMROD-W statistical software (design NEMROD-W, version 9901 Française, LPRAI-Marseille Inc., France) (https://www.nemrodw.com/fr).

When grown on mineral medium ONR7a supplemented with crude oil and vegetable oil as sole carbon sources, BUN14 strain produced biosurfactants, as indicated by the oil spreading, drop collapse and emulsification tests (Fig. S2). Use of the Cetyl Tri Ammonium Bromide (CTAB)-Methylene blue agar method indicated that BUN14 produced anionic biosurfactant (Fig. S2). Optimal conditions for growth and biosurfactant production were assessed using response surface methodology (RSM) experiments. The analysis of variance for the fitted mathematical models shows that the regression sum of squares was statistically significant (P < 0.01) (Table S5). Response surface plots, showing optimal biosurfactant production conditions, are shown in Fig. 1. Statistical analyses of central composite design (CCD) experiments demonstrated that the percentage of oil substrate, NaCl concentration, inoculum size and incubation time all affected the biosurfactant production. Based on desirability function, the optimal BUN14 growth (OD_600nm_, 0.54), biosurfactant production (OD_625nm_, 2.58) and activities (emulsion index E24, 40.09% and oil-displacement ODA, 20.40 cm^2^), were obtained after approx. 7 days of cultivation crude oil, 2.50% NaCl concentration inoculum size source (Fig. S3). Accordingly, as for previous studies^20,21^ substrate, NaCl concentration, inoculum size and incubation time significantly affected biosurfactant production (0.01 < p < 0.05).

**Figure 2:**
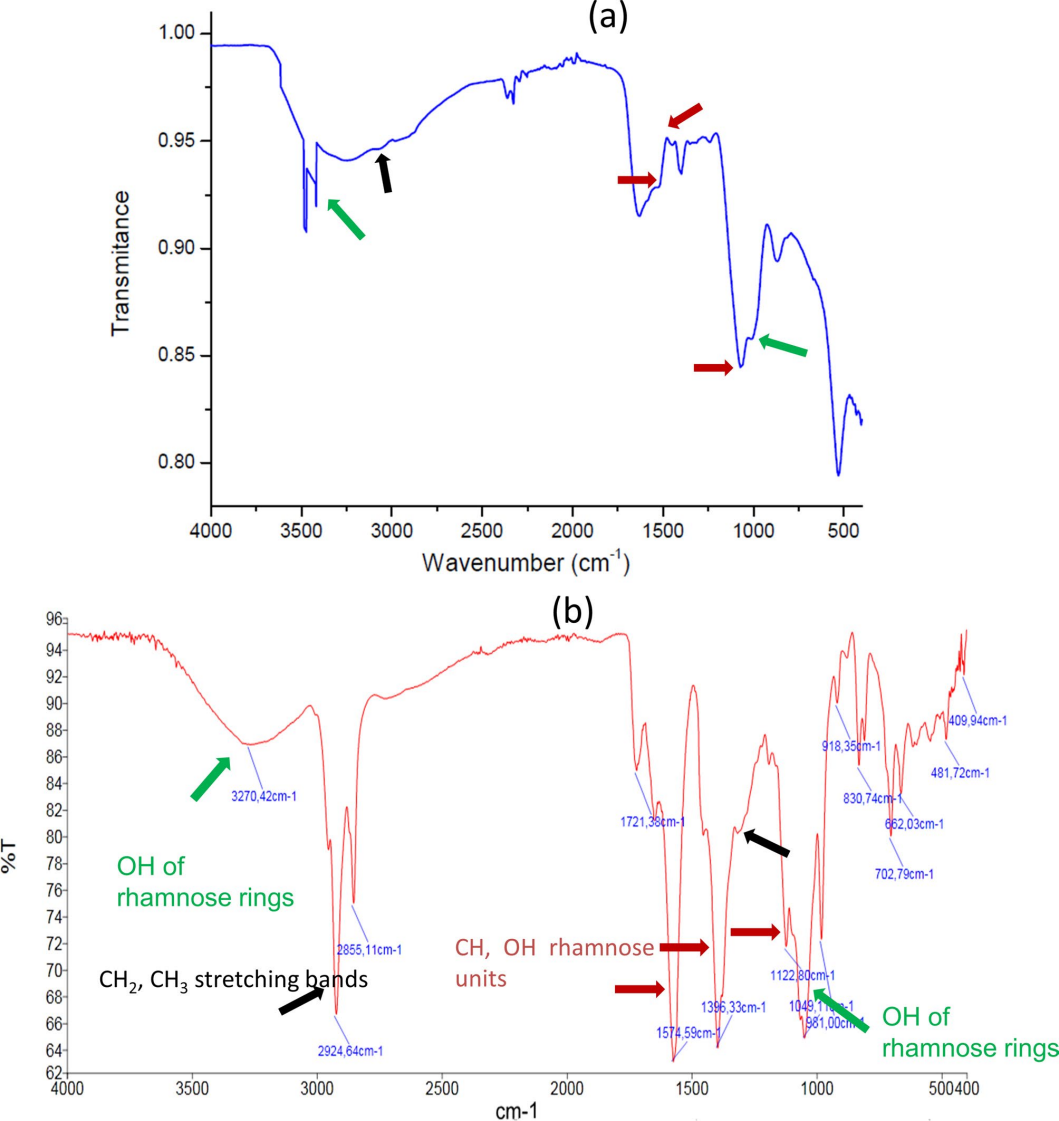
Fourier transform infrared spectroscopy spectra of (**a**) obtained biosurfactant of BUN14 (**b**) a commercial *Pseudomonas aeruginosa* rhamnolipid BS (R90) (AGAE Technologies, Corvallis, OR, USA) used as a reference standard.

The basic structure of the isolated biosurfactant was evaluated by Fourier Transform InfraRed (FT-IR) spectrometry and compared to a reference *Pseudomonas aeruginosa* (Fig. 2). The peaks appearing at 3278.56 (Fig. 2a) and 3270.42 cm^−1^ (Fig. 2b) denoted the presence of –OH stretching (free hydroxyl groups of rhamnose rings) of hydroxyl group. The adsorption peaks at 2924.64 (Fig. 2b) and 3000.0 cm^−1^ (Fig. 2a) indicated the presence of terminal methyl group of aliphatic stretching bands (CH2, CH3). The absorption peaks at 1097.35 (Fig. 2a) and 1049.1 cm^−1^ (Fig. 2b) confirmed the presence of C–O–C vibrations (rhamnose ring). The area between 1495.92 and 1150.02 cm^−1^ (Fig. 2a) represented C–H and OH deformation vibrations typical for carbohydrates, as in the rhamnose units of the biosurfactant. The apparent similarity of the main functional groups determined by FTIR spectrometry of the commercial rhamnolipid (R90) from *Pseudomonas aeruginosa* (AGAE Technologies, Corvallis, OR, USA) and the isolated biomolecule from strain BUN14 (Fig. 2), suggested that the BUN14 biosurfactant product was composed on rhamnose rings with long hydrocarbon chains.

Members of the genus *Pseudomonas* are known for biosynthesis of rhamnolipids with biosurfactant properties^20,22^.

### Hydrocarbon degradation

Strain BUN14 could grow on and utilize various hydrocarbons, including pyrene, naphthalene, phenanthrene, carbazole, dibenzofuran, dibenzothiohene, biphenyl, pristane, fluoranthene, crude oil, octadecane and tetradecane as sole carbon and energy sources (Fig. S4a–c). These findings are in agreement with previous reports demonstrating that many *Pseudomonas* species are capable of utilizing hydrocarbons as carbon and energy sources^23–25^. The kinetics of hydrocarbon biodegradation in liquid media was determined using GC-FID analysis. The total degradation of petroleum TERHCs by strain BUN14 was estimated at 90% after 21 days with rapid degradation of almost all alkanes C_12_–C_36_ (Fig. S4d,e). Of the more recalcitrant aliphatic compounds, 22%, 40%, 30% and 14% of pristane, naphthalene, DBT and DBF, respectively, were degraded by strain BUN14 (Fig. S4d) over a period of 21 days. The ability of various bacteria to degrade naphthalene, including *Sphingomonas, Pseudomonas* and *Acidovorax,* has been reported^24, 26–29^.

### General features of the draft genome of strain BUN14

The draft genome sequence of strain BUN14 was assembled into 22 contigs of 4,898,053 bp in size with a G + C content of 62.4%. Comparisons of the BUN14 genome with those of its three closest relatives, *P. kunmingensis* DSM 25974, *P. kunmingensis* CCUG 36651 and *P. chloritidismutans* AW-1, showed similar genome sizes and G + C contents range of 4.65–5.06 Mb and 62.4–62.6%, respectively (Fig. S5, Table 1).

**Table 1:** Genomic comparison with related *Pseudomonas* strains obtained from the NCBI database.

Strains	Accession	Assembly	# contigs	Size (bp)	G+C (%)	# proteins	# tRNA	*completeness (%)
*Pseudomonas kunmingensis* DSM 25974^T^	GCF_900114065.1	IMG-taxon 2663763606	33	4,654,543	62.6	4341	55	99.6
*Pseudomonas chloritidismutans* AW-1	GCF_000495915.1	PseChl	77	5,056,349	62.5	4719	85	96.9
*Pseudomonas kunmingensis* CCUG 36651	GCF_002890855.1	ASM289085v1	65	4,810,086	62.4	4441	55	99.8
*Pseudomonas kunmingensis* BUN14	GCF_002929225.1	ASM292922v1	22	4,898,053	62.4	4552	55	99.8
*Pseudomonas songnenensis* NEAU-ST5-5^T^	GCF_003696315.1	ASM369631v1	25	4,321,177	63.2	4021	58	100
*Pseudomonas stutzeri* ATCC 17588^T^	GCF_000219605.1	ASM21960v1	1	4,547,930	63.9	4191	65	99.6
*Pseudomonas balearica* DSM 6083^T^	GCF_000818015.1	ASM81801v1	1	4,383,480	64.7	4060	64	99.7
*Pseudomonas saudiphocaensis* 20_BN^T^	GCF_000756775.1	PRJEB6478_assembly_1	1	3,673,759	61.2	3414	54	99.8
*Pseudomonas zhaodongensis* NEAU-ST5-21^T^	GCF_003696365.1	ASM369636v1	27	4,668,208	59.6	4293	58	100
*Pseudomonas xanthomarina* DSM 18231^T^	GCF_900129835.1	IMG-taxon 2687453778	17	4,308,853	60.3	3968	54	99.5
*Pseudomonas azotifigens* DSM 17556^T^	GCF_000425625.1	ASM42562v1	59	5,017,423	66.7	4520	55	99.8
*Pseudomonas flexibilis* ATCC 29606^T^	GCF_000802425.1	ASM80242v1	49	3,762,694	65.8	3503	62	99.4
*Pseudomonas oryzae* KCTC 32247^T^	GCF_900104805.1	IMG-taxon 2667527434	1	4,642,193	67.4	4130	87	99.5
*Pseudomonas linyingensis* LMG 25967^T^	GCF_900109175.1	IMG-taxon 2663762776	43	4,721,122	66.3	4308	62	100
*Pseudomonas sagittaria* JCM 18195^T^	GCF_900115715.1	IMG-taxon 2663762798	44	4,607,575	66.7	4164	68	100
*Pseudomonas fluvialis* ASS-1^T^	GCF_002234375.1	ASM223437v1	69	3,291,143	62.6	3049	56	99.3
*Pseudomonas caeni* DSM 24390^T^	GCF_000421765.1	ASM42176v1	24	3,022,325	48.3	2781	43	99.1

Annotation of the strain BUN14 genome yielded 4551 protein coding sequences (CDSs) and 55 tRNA genes (Table S1). Evaluation of the predicted proteins using BUSCO showed that 99.8% of gammaproteobacteria conserved single copy orthologs were present in the strain BUN14 draft genome with 0 duplication (Table 1). Functional annotation of the predicted proteome showed that ~ 87% of the proteins could be assigned to EggNOG orthologous genes (OGs). The overall distribution revealed that the category *metabolism* was overrepresented, comprising ~ 35% of the OGs compared to cellular processes and signalling, and information storage and processing, which comprise ~ 23% and 17%, respectively (Table S1).

### Phylogenomic comparison of strain BUN14 and related *Pseudomonas* strains

**Figure 3:**
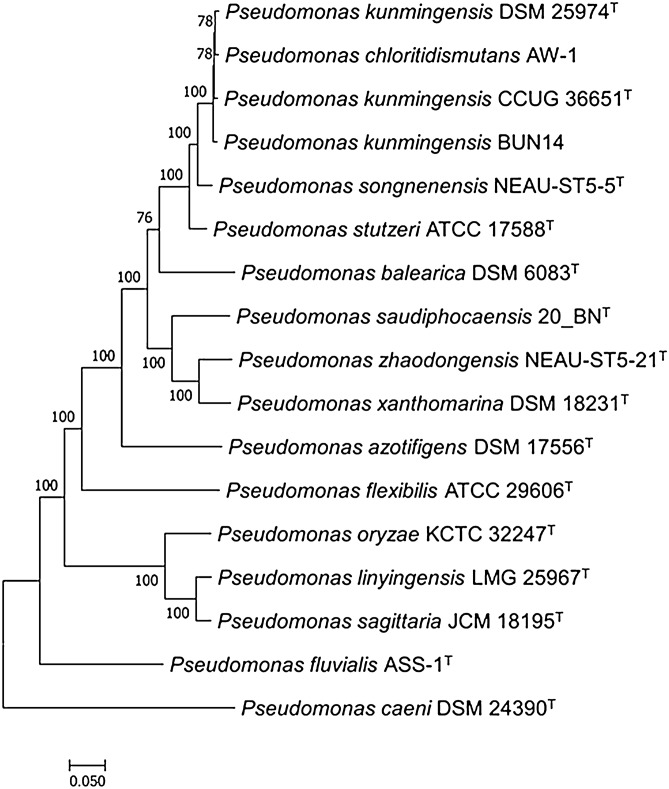
Phylogenomic analysis of strain BUN14 and 16 closest *Pseudomonas* type strains*.* The maximum likelihood (ML) tree was inferred from the concatenated protein alignment (376,464 amino acids) of 1238 single copy proteins. The phylogeny was generated using IQ-TREE version 1.6.7 based on the LG + F + R5 model with the bootstrap option-bb 1000.

To identify appropriate comparison strains, 189 core single copy proteins from 294 *Pseudomonas* Type Strains were used to build a guide tree for subsequent phylogenomic analysis (Fig. 3). Strain BUN14 grouped distinctly with 14 *Pseudomonas* Type Strains, including *P. kunmingensis* HL22-2^T^. Phylogenetic analysis based on 1,238 single copy proteins shared among the *Pseudomonas* strains that cluster with strain BUN14 and *P. caeni* DSM 24390^T^, included as an outgroup, showed hat strain BUN14, *P. kunmingensis* DSM 25974^T^, *P. chloritidismutans* AW-1 and *P. kunmingensis* CCUG 36651 formed a subclade, distinct from *P. songnenensis* NEAU-ST5-5^T^ and *P. stutzeri* ATCC 17588^T^ which were included in two separate clades (Fig. S6). Strain BUN14, DSM 25974^T^, AW-1 and CCUG 36651 shared an average nucleotide identity (ANI) range of 96.66–97.18% (Fig. S7). By contrast, the four strains shared ANI ranges of 86.74–86.88% with *P. stutzeri* ATCC 17588^T^. Furthermore, AW-1 and DSM 25974^T^ shared in silico DNA-DNA hybridization (*i*DDH) of 71.9% (CI 68.9–74.8%) while strain BUN14 shared *i*DDH of 72.2% (CI 69.2–75.1%) and 70.3% (CI 67.3–73.2%) with AW-1 and DSM 25974^T^, respectively (Fig. S8). Two important species discriminatory genomic metrics, ANI and *i*DDH, provided further support for the clustering of these strains. Interestingly, the two type strains (DSM 25974T and AW-1), strains BUN14 and CCUG 36651 share ANI and *i*DDH values above 96 and 70%, respectively, suggesting that they belong to the same species^30–33^. The ANI range of 86.74–86.88% between the four strains and *P. stutzeri* ATCC 17588^T^ and the distinct clustering of the latter in the presented phylogeny provide strong evidence for the separation of the above four from the *P. stutzeri* group. Mehboob et al.^17^ reported similar ANI values to the current data for AW-1 and strains *P. stutzeri* except *P. stutzeri* CCUG 29243 which share ANI value of 97% with AW-1. However, our work included only the type strain of *P. stutzeri*. *P. chloritidismutans* AW-1. The latter was first described as a novel species in 2002 based on DDH, physiological and biochemical data^14^ and later proposed as conspecific with *P. stutzeri*, specifically *P. stutzeri* genomovar 3, based on multigenic phylogeny and by disputing the validity of the phenotypic features that separate the two taxa^34^. Subsequent studies^17^ and this work clearly demonstrate that *P. chloritidismutans* AW-1 and *P. stutzeri* ATCC 17588^T^ are distinct species^34^. *P. kunmingensis* DSM 25974^T^^35^ was proposed as a distinct species based on a combination of 16S rRNA gene analysis, phenotypic characterisation and DDH values^13^. However, the analysis omitted *P. chloritidismutans* AW-1 from the comparison. For instance, the low 16S rRNA gene similarity value reported between DSM 25974^T^ and closely related taxa was only possible without AW-1. The full-length 16S rRNA genes of AW-1 and DSM 25974^T^ (data not shown), extracted from their respective genomes, share a similarity of 99.74%. Moreover, the phylogenetic and the genomic metrics presented here indicate that DSM 25974^T^ and AW-1 share ANI and DDH values that are below the threshold for species separation^36–38^. Considering the priority of *P. chloritidismutans* AW-1^T^ and the multiple lines of phylogenetic and genomic evidence, we suggest that *P*. *kunmingensis* DSM 25974^T^ is a heterotypic synonym of *P. chloritidismutans* AW-1^T^. Similarly, the strains BUN14 and CCUG 36651, and perhaps CCUG 29243^17^, may be more appropriately affiliated with *P. chloritidismutans*.

### Genomic determinant of hydrocarbon degradation in strain BUN14 and comparisons with closely related *Pseudomonas* spp

To identify the proteins potentially implicated in hydrocarbon degradation, the proteome of strain BUN14 was annotated using BlastKOALA^37^. Of the 4551 proteins, 2564 (56.34%) could be assigned to 2062 KEGG Orthologues (KOs) including 154 linked to xenobiotic biodegradation and metabolism (Fig. S9). Of the 154 proteins, 44 have been associated with the pathways for complete degradation of benzoate and naphthalene (Table S2). Gene product prediction using FGENESB revealed that 39 of these proteins are encoded in four genomic loci, comprising five putative operons, while the genes of five proteins appear to be transcribed as independent transcription units.

**Figure 4:**
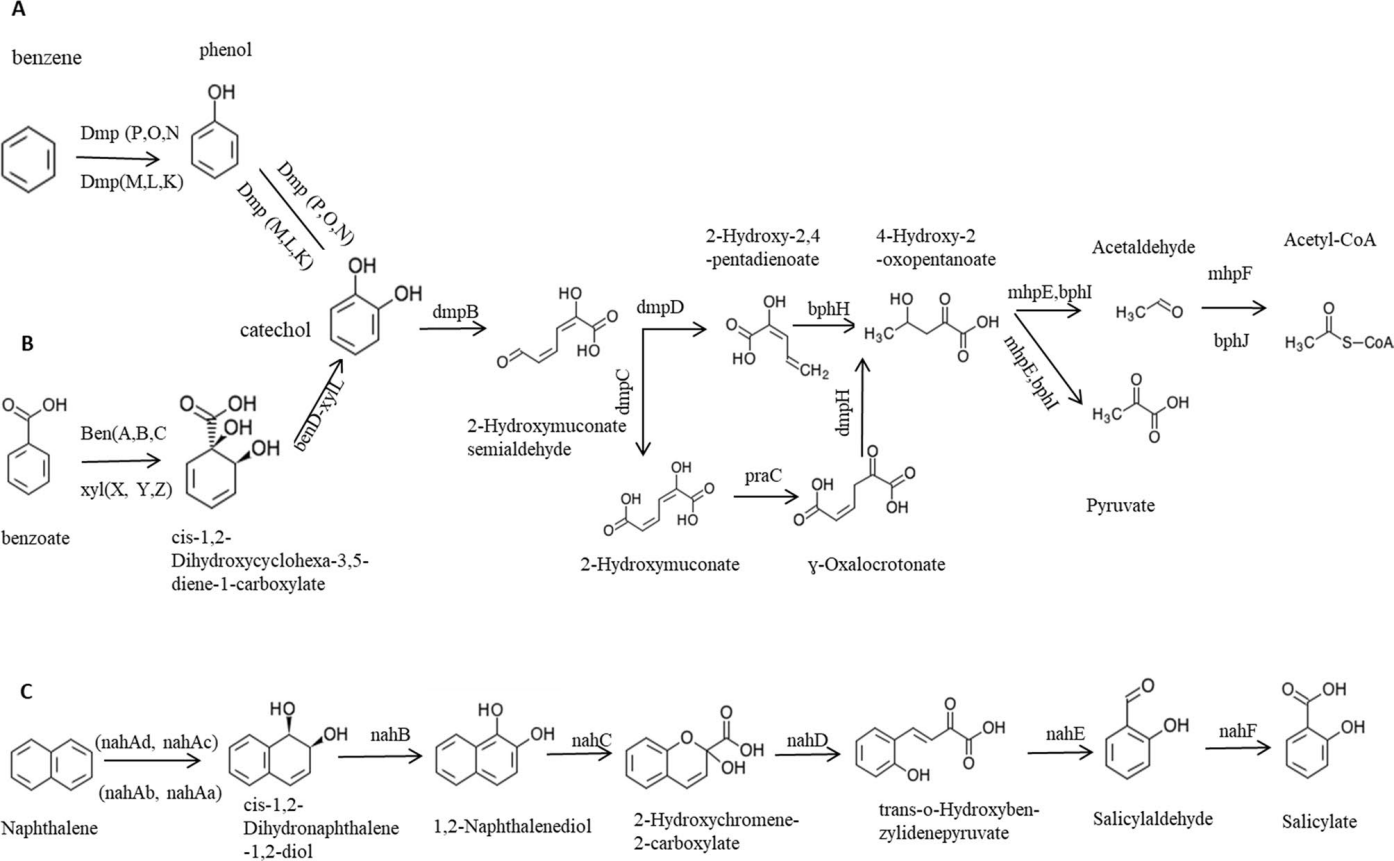
Benzoate (**A**), benzene (**B**) and naphthalene (**C**) pathways degradation in BUN14.

The strain BUN14 genome contained six genes (*dmpP, dmpO, dmpN, dmpM, dmpL* and *dmpK*) encoding enzymes that catalyse the conversion of benzene to catechol (KEGG module: M00548; Fig. 4). The genome also contained the genes *benA-xylX, benB-xylY, benC-xylZ* and *benD-xylL*, which encode the enzymes for degradation of benzoate to catechol via catechol/methylbenzoate (KEGG module: M00551) (Fig. 4). Two distinct genomic loci contained *bphH, bphI, bphJ, dmpB, dmpC, dmpD, dmpH, mhpE, mhpF* and *praC,* encoding enzymes for the complete pathway for degradation of catechol (KEGG module: M00569, meta-cleavage) to yield Acetyl-CoA (Fig. 4), and the *catB, catC, catA*, and *pcaD* genes that encode enzymes for conversion of catechol (KEGG module: M00568; ortho-cleavage) to 3-oxoadipate (Fig. 4). The complete degradation pathway of benzoates and benzoate derivatives, yielding acetyl-CoA and pyruvate, has been identified in other *Pseudomonas* species such as *Pseudomonas putida, Pseudomonas aeruginosa and Pseudomonas alcaliphila*^8,23,39^.

Although partial degradation of dioxin was detected in culture experiments and the analysis of genome showed only some of genes of the dioxin degradation pathway; *praC, dmpH bphI, bphH, bphJ, mhpE* and *mhpF* (Table S2), associated with the conversion of 2-hydroxymuconate to acetyl-CoA. However, *dxnA/dbfA, dbfB and dxnB* which encode enzymes involved in the conversion of dibenzo-p-dioxin to 2-hydroxymuconate have not been identified. Genomic analysis of *Pseudomonas putida* strain B6-2 revealed genes for the complete biphenyl degradation pathway (represented by the gene cluster b*phA-D and pbhH-K*)^40^. Like strain BUN14, the genome sequence of *Sphingomonas. wittichii* contains only putative genes that code for enzymes in the initial part of the dioxin degradative pathway^41^.

**Figure 5:**
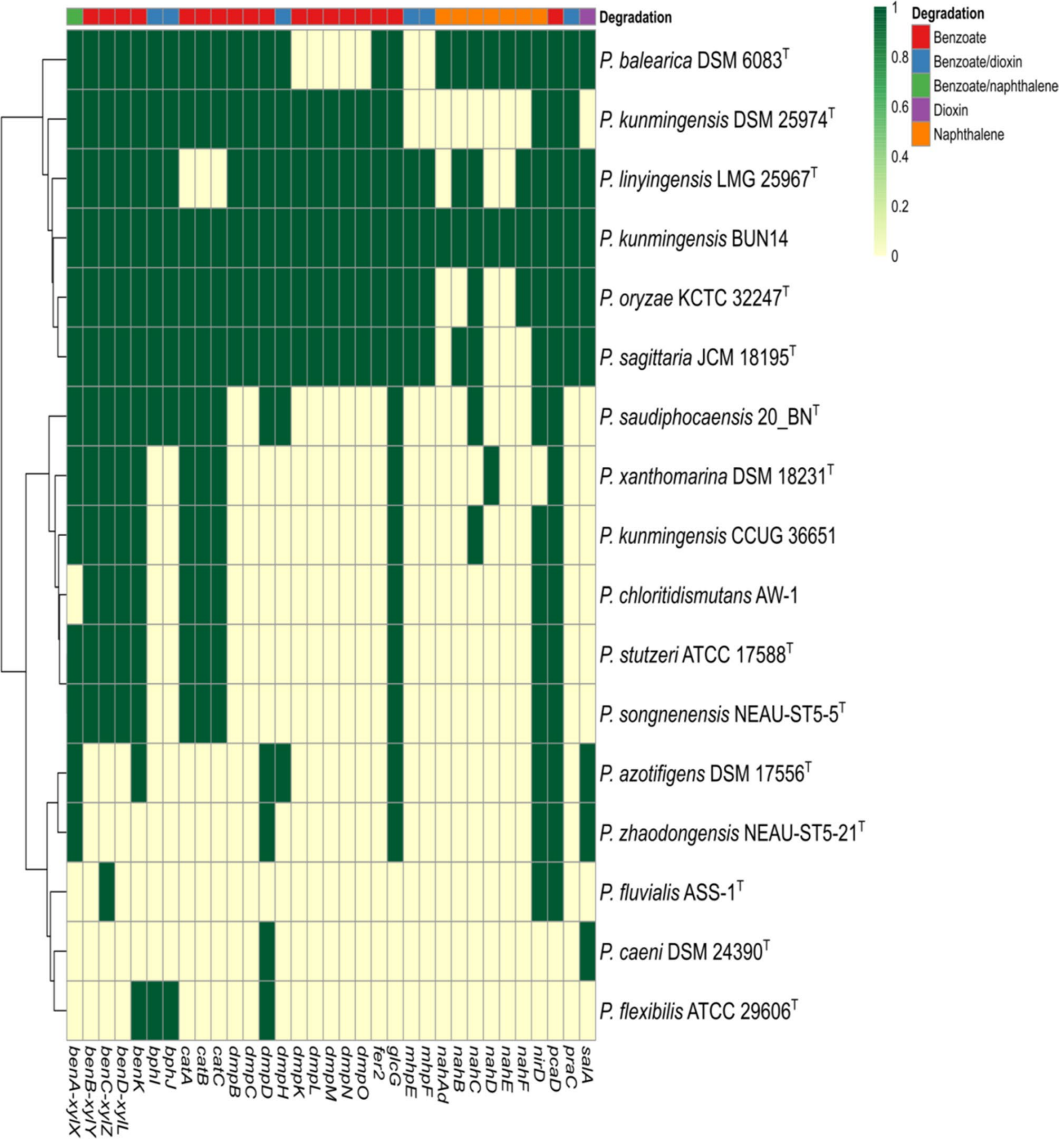
Heat map showing the distribution of orthologous proteins of strain BUN14 and closely related species, associated benzoate, dioxin and naphthalene degradation pathways. The heat map was based on the presence/absence matrix of orthologous proteins determined using Orthofinder. Heat map was produced using Clustvis version 1 (https://biit.cs.ut.ee/clustvis/).

**Figure 6:**
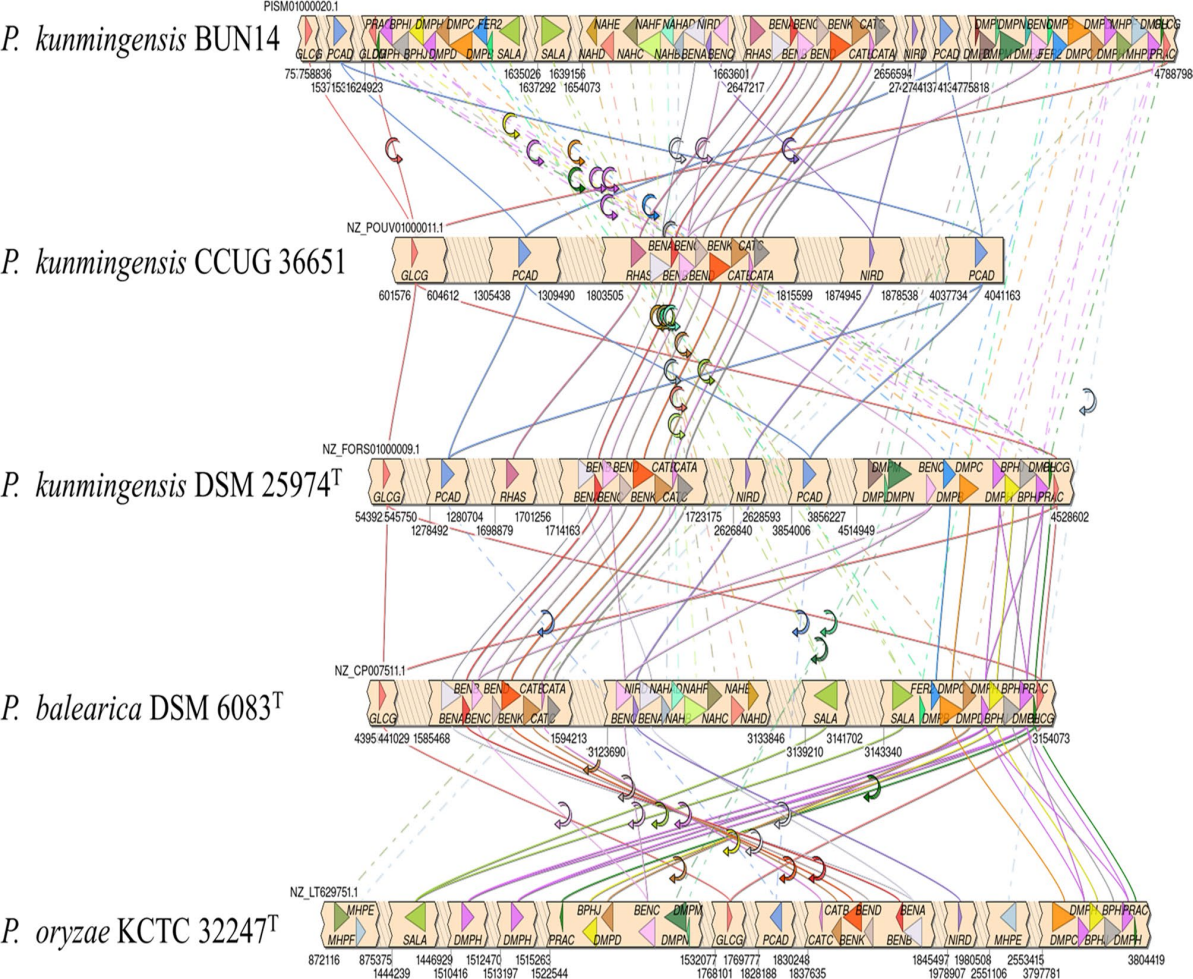
Synteny map showing the genomic locations of genes linked to benzoate, dioxin and naphthalene degradation pathways for strain BUN14 and related species. The map was generated with SimpleSynteny server version 1.4 (https://dveltri.com/simplesynteny/advanced.html).

The strain BUN14 proteome included the nine proteins (nahD, nahE, nahC, nahF, nahB, nahAd, nahAc, nahAb and nahAa) that catalyse the complete pathway for the degradation of naphthalene (KEGG module: M00534) to salicylate (Fig. 5). Comparisons of the strain BUN14 genome with those of 15 related *Pseudomonas* species, including its closest relatives, *P. kunmingensis* DSM 25974^T^, CCUG 36651 and *P. chloritidismutans* AW-1, showed that only BUN14 and *P. balearica* DSM 6083T harboured the complete pathway for naphthalene degradation (Fig. 6). Apart from DSM 18231T, which has an orthologue of nahD, all the compared strains lacked the orthologues of nahD and nahE. However, DSM 18231T lacked other genes associated with the naphthalene degradation pathway, and the other *Pseudomonas* genomes lacked between two and all nine genes of the complete pathway. The catechol degradation pathway has been shown to be the major catabolic route by which many bacteria biodegrade naphthalene^42^ and a repertoire of genes linked to the compete naphthalene degradation pathway has been reported in many taxa, including *Pseudomonas, Paraburkholderia, Alcaligenes and Rhodococcus*^23,42–45^. The BUN14 genome encoded a membrane bound acyl-CoA desaturase (WP_104098302.1; FADS-like; alkB like), rubredoxin-2 (WP_003283319.1) and rubredoxin-NAD(+) reductase (WP_104098084.1), which are likely to determine its ability to degrade n-alkane. Orthologues of these three proteins were also found in the proteomes of nine of the compared genomes including the closest relatives of BUN14, *P. kunmingensis* DSM 25974^T^, CCUG 36651 and *P. chloritidismutans* AW-1 (Fig. S10). Unlike *P. chloritidismutans,* no alkane 1-monooxygenase (AlkB; WP_023446487.1) genes were identified in the strain BUN14 genome. Most AlkB proteins have been shown to carry membrane fatty acid desaturase (FADSs), the actions of which depend on rubredoxin and rubredoxin reductase, encoded on a distinct genomic region^10^. Both rubredoxin and rubredoxin reductase homologues were identified in the BUN14 genome, suggesting that either an alternative, and unknown, enzyme catalyses the initial activation of alkanes, or that the sequence homology of the BUN14 putative AlkB gene was too low to be detected by Blast analysis.

Given the identification of putative rhamnolipids in the extracted biosurfactant fraction of BUN14 cultures, the proteome was scanned for proteins potentially involved in the synthesis of this group of compounds. Blastp searches against 3-(3-hydroxydecanoyloxy) decanoate synthase RhlA (Q51559), rhamnosyl transferase RhlB (Q51560) and Rhamnosyltransferase 2 RhlC (Q9I4K5) of *P. aeruginosa* did not yield orthologous of these proteins. The BUN14 proteome was subsequently annotated for glycosyltransferase (GT1 and GT2) signatures using dbCAN2^46^ and for conserved domains using the NCBI conserved domains database search tool^47^. This combined strategy revealed four proteins (Table S3) that showed similar domain architecture to *P. aeruginosa* RhlA, RhlB and RhlC. WP_104098192.1 (296 aa) which shared only 13.6% similarity with Q51559 (295 aa) of *P. aeruginosa*, harboured conserved domains similar to Q51559 and was identified as a specific hit for the alpha/beta hydrolase superfamily.Only one BUN14 protein, WP_104098902.1 (310 aa), was predicted to be a member of the glycosyltransferase family GT1. This protein shared 22.1% similarity with Q51560 (325 aa) of *P. aeruginosa* and the domain architecture of both proteins was similar. Seven BUN14 proteins were predicted to be members of the glycosyltransferase family GT2. Of these, two hypothetical proteins, WP_042926909.1 (291 aa) and WP_003300529.1 (357 aa) were predicted to harbour functional domains similar to those of Q9I4K5 (325 aa) of *P. aeruginosa*. However, WP_042926909.1 and WP_003300529.1 share only 20.5 and 22% similarity, respectively, with Q9I4K5. Therefore, WP_104098192.1 and WP_104098902.1 could potentially carry the molecular functions of the RhlA and RhlB proteins, respectively, while WP_042926909.1 and WP_003300529.1 are potential RhlC functional homologues. Indeed, the genetic characterization of rhamnolipid biosynthesis pathways is hindered by high levels of sequence diversity in the key genes, as seen in the low levels or absence of homology in the *rhl*A-C genes/proteins in *P. aeruginosa*, *Burkholderia* and related organisms^48^.

## Conclusion

This study has demonstrated the versatility of *Pseudomonas* strain BUN14 in the degradation of a wide range of polyaromatic and aliphatic hydrocarbons. The genome of strain BUN14 was fully sequenced to identify the genetic determinants of its functional capacity, including the ability to utilize various hydrocarbons and to produce biosurfactants. The complete degradation pathways of some hydrocarbons such as benzoate and naphthalene were identified in the BUN14 genome, while the absence of some key enzyme genes suggested that the genome may harbor genes encoding novel functionalities, or functional homologues with very low sequence homology. On the basis of whole genome phylogeny and other genomic metrics (ANI and iDDH), the current study demonstrates that *P. chloritidismutans* AW-1^T^ is the closest relative of strain BUN14. Furthermore, *P. chloritidismutans* AW-1^T^ and *P. kunmingensis* DSM 25974 are conspecific and given that the former has been validly described prior to *P. kunmingensis* DSM 259, we suggest that DSM 25974 strain should be considered as a heteronym of *P. chloritidismutans* AW-1^T^.

## Materials and methods

### Strain isolation and carbon source utilization

Strain BUN14 was isolated by enrichment culture in mineral salts medium (ONR7a) supplemented with 1% crude oil as a sole carbon source, after incubation for 21 days at 30 °C with shaking (150 × g)^5^. ONR7a contained (per liter of distilled or deionized water) 22.79 g of NaCl, 11.18 g of MgCl_2_·6H_2_O, 3.98 g of Na_2_SO_4_, 1.46 g of CaCl_2_–2H_2_O, 1.3 g of TAPSO {3-[N-tris(hydroxymethyl) methylamino]-2-hydroxypropanesulfonic acid}, 0.72 g of KCl, 0.27 g of NH_4_Cl, 89 mg of Na_2_HPO_4_·7H_2_O, 83 mg of NaBr, 31 mg of NaHCO3, 27 mg of H_3_BO_3_, 24 mg of SrCI·6H_2_O, 2.6 mg of NaF, and 2.0 mg of FeCl_2_·4H_2_0. 1% of crude oil was used as only energy and carbon source^45,49^. To determine the phylogenetic affiliation of the strain, the 16S rRNA gene was amplified, sequenced, and queried using the 16S rRNA identification module in EzBioCloud^18,19^. The 16S rRNA sequences of the closest type strain relatives of BUN14 obtained from the Ezbiocloud database were aligned using Mafft^50^. Gaps were removed using the default setting in Gblocks v.0.91b^51^. A phylogenetic tree was constructed by the neighbour joining method and the tree topology was evaluated by performing bootstrap analysis of 1000 data sets using MEGA6.0^52^.

The cellular morphology of BUN14 was visualized using a JCM-5700 Scanning Electron Microscope, resolution 0.6 nm, specimen size 5 mm ∅ × 0.6 mm high, with a Gatan Digital Micrograph imaging system and SE & BS detectors^53^. BUN14 cultures were centrifuged (10 min 14,000*g*) and bacterial biomass was fixed in 2.5% gluteraldehyde in 0.075 M K-phosphate buffer (pH 7.4) for 2 h at room temperature. The preparation of the Scanning Electron Microscope (SEM) was performed according to Stanton et al.^53^. To test the ability of strain BUN14 to utilize hydrocarbon substrates as the sole carbon and energy source, the organism was inoculated onto solid ONR7a agar media containing individual hydrocarbons (pristane, phenanthrene, pyrene, naphthalene, fluoranthene, dibenzothiophene, dibenzofuran, squalene, and carbazole: 50 mM final concentration) and incubated for 7 days at 30 ± 1 °C. Growth of bacterial colonies was considered as a positive indication of the capacity to use the substrate as the sole carbon and energy source.

### Optimization of surfactant activities

Strain BUN14 was screened for biosurfactant production using the Cetyl Trimethyl Ammonium Bromide agar plate assay (CTAB), the drop collapse test, emulsion index (E_24_) and the oil-displacement test (ODA)^5,54,55^. For the optimization of surfactant activities, response surface methodology (RSM) using central composite design (CCD) was performed (Table S4). A CCD composed of 29 experiments have been planned (Table S5). The effects of various independent variables (substrate (waste frying oil) concentration (X1), NaCl concentration (X2), inoculum size (X3) and incubation time (X4)) on the biosurfactant production yield (Response Y) were evaluated at three levels (Table S6). The specific codes for each independent variable and range are given in Table S6. The relationship between these variables and the biosurfactant production yield was defined by the following model:

$$\begin{aligned} {\text{Y}} & = {\text{b}}_{0} + {\text{b}}_{{1}} {\text{X}}_{{1}} + {\text{b}}_{{2}} {\text{X}}_{{2}} + {\text{b}}_{{3}} {\text{X}}_{{3}} + {\text{b}}_{{4}} {\text{X}}_{{4}} + {\text{b}}_{{{11}}} {\text{X}}_{{1}}^{{2}} + {\text{b}}_{{{22}}} {\text{X}}_{{2}}^{{2}} + {\text{b}}_{{{33}}} {\text{X}}_{{3}}^{{2}} + {\text{b}}_{{{44}}} {\text{X}}_{{4}}^{{2}} + {\text{b}}_{{{12}}} {\text{X}}_{{1}} {\text{X}}_{{2}} + {\text{b}}_{{{13}}} {\text{X}}_{{1}} {\text{X}}_{{3}} + {\text{b}}_{{{23}}} {\text{X}}_{{2}} {\text{X}}_{{3}} \\ & \quad + {\text{b}}_{{{14}}} {\text{X}}_{{1}} {\text{X}}_{{4}} + {\text{b}}_{{{24}}} {\text{X}}_{{2}} {\text{X}}_{{4}} + {\text{b}}_{{{34}}} {\text{X}}_{{3}} {\text{X}}_{{4}} . \\ \end{aligned}$$

where Y are the response (emulsion index (*E*_24_) and oil displacement activity (ODA); Xj is the variables of studied factors and b_0_, b_j_, b_jk_, and b_jj_: model coefficients.

The five replicates at the center point were carried out in order to estimate the pure error variance. The significance of the fitted model was tested by the means of the analysis of variance (ANOVA). The relationship between the response and the experimental variables was illustrated graphically by plotting the response surfaces. The NemrodW software was used for experimental design and statistical analysis^56^. The optimum conditions for maximum biosurfactant production was determined using the desirability functions^57^.

The extraction of the biosurfactant was performed using liquid–liquid extraction^20^. The supernatants of BUN14 cultures were collected after centrifugation (12,000 rpm/20 min at 4 °C) adjusted to pH 2.0 with 6 N HCl and left overnight at 4 °C. The precipitates surfactant was collected by centrifugation at 12,000 rpm for 30 min at 4 °C. For additional purification, the crude biosurfactant was extracted at three successive washes with a mixture of the chloroform–methanol (2:1, v/v) and concentrated using rotary evaporation at 40 °C^20^. Functional group evaluation of the extracted biosurfactant were performed using Fourier Transform Infra-Red spectroscopy (Perkin Elmer FTIR model 2000) as described previously^20,58^. Infrared absorption spectra were obtained over the range of 400–4000 cm^−1^ with a resolution of 4 cm.

### Hydrocarbon degradation

For hydrocarbon degradation analysis, strain BUN14 was cultured in ONR7a liquid mineral medium supplemented with 5 g L^−1^ Na-acetate for 72 h in shaking (150 × g) at 30 ± 1 °C. Bacterial cells in logarithmic phase were collected by centrifugation (10 min, 14,000*g*), washed twice in phosphate buffered saline (PBS 1×; 140 mM NaCl, 2.7 mM KCl, 4.3 mM Na_2_HPO_4_·7H_2_O and 1.5 mM KH_2_PO_4_), resuspended and inoculated (~ 10^6^ cells ml^−1^ measured by the DAPI count method) into 50 ml ONR7a liquid mineral medium supplemented with sterile Arabian Light Crude Oil (1%, v/v), pristane (1% v/v) and 50 ppm (final concentration) of naphthalene, DBT and DBF (Sigma Aldrich, Milano—Italy). Cultures containing the same amount of hydrocarbon but without inoculation were used as abiotic controls. Cultures were incubated at 30 ± 1 °C for 21 days with shaking. Total Extracted and Resolved Hydrocarbons and their derivatives (TERHCs) were extracted from cultures using dichloromethane (Sigma-Aldrich, Milan; 10% v/v) following the 3550C EPA (Environmental Protection Agency) procedure as previously reported^59,60^. Degradation rates were quantified by a Master GC DANI Instruments GC-FID (Development ANalytical Instruments DANI Instruments S.p.A., Milan, Italy), equipped with SSL injector and FID detection. The extent of biodegradation was expressed as the percentage of hydrocarbon degraded compared to the abiotic control^60^.

### BUN14 genome sequencing, assembly, annotation and analysis

DNA extraction was performed on mid-log phase cells by sodium dodecyl sulfate (SDS)-proteinase K treatment with an additional equal volume of chloroform/isoamyl alcohol (24:1 v/v). Purified genomic DNA was sequenced on an Illumina MiSeq platform (MRDNA, Clearwater, Tx, USA). The 11,335,376 paired reads were filtered according to read quality, and reads below a mean quality, score of 23 were removed using prinseq-lite software. The reads were assembled using SPAdes^61^. The genome of strain BUN14 was structurally annotated using PROKKA^62^ and FGENESB (http://www.softberry.com/). The CGView Server^63^ was used for circular representation of multiple genomes. The draft genome of strain BUN14 was used as the reference genome and was compared with genomes of *P. kunmingensis* DSM 25974, *P. kunmingensis* CCUG 36651 and *P.chloritidismutans* AW-1. Functional annotation was accomplished using a combination of RAST^63^, BlastKOALA^37^, KEGG^64,65^ and EggNOG^66^. Orthologous relationships among the predicted proteomes of strain BUN14 and close relatives were determined using orthofinder^67^. Single copy orthologues were aligned using mafft^50^, trimmed using Gblocks v.0.91b^51^ and maximum likelihood phylogeny was constructed using iq-tree^68^. Orthologous average nucleotide identity (OrthoANI) and in *silico* DNA-DNA hybridization (*i*DDH) were determined using OAT^69^ and GGDC^38^, respectively. Comparisons of genomic regions among related *Pseudomonas* species were accomplished using SimpleSynteny^70^ and a heat map was generated using ClustVis^71^.

### Nucleotide sequence accession number

The genome sequence has been deposited in the Bioproject and Biosample Genomes online database under PRJNA420855 and SAMN08122818 accession numbers, respectively.

Whole Genome Shotgun sequence data has been deposited at DDBJ/ENA/GenBank under the accession number PISM00000000.

## Supplementary Information

Supplementary Information.

## Data Availability

The genome sequence has been deposited in the Bioproject and Biosample Genomes online database under PRJNA420855 and SAMN08122818 accession numbers, respectively. Whole Genome Shotgun sequence data has been deposited at DDBJ/ENA/GenBank under the accession number PISM00000000.

## References

[1] McKee RH, Adenuga MD, Carrillo J-C (2015). Characterization of the toxicological hazards of hydrocarbon solvents. Crit. Rev. Toxicol..

[2] Van den Berg M (2006). The 2005 World Health Organization reevaluation of human and mammalian toxic equivalency factors for dioxins and dioxin-like compounds. Toxicol. Sci..

[3] Head IM, Jones DM, Röling WF (2006). Marine microorganisms make a meal of oil. Nat. Rev. Microbiol..

[4] Mahjoubi, M., Cappello, S., Souissi, Y., Jaouani, A. & Cherif, A. Microbial bioremediation of petroleum hydrocarbon–contaminated marine environments. in *Recent Insights in Petroleum Science and Engineering* (ed. Zoveidavianpoor, M.) 325–350. 10.5772/intechopen.72207 (2018).

[5] Mahjoubi M (2013). Hydrocarbonoclastic bacteria isolated from petroleum contaminated sites in Tunisia: Isolation, identification and characterization of the biotechnological potential. New Biotechnol..

[6] Yakimov MM, Timmis KN, Golyshin PN (2007). Obligate oil-degrading marine bacteria. Curr. Opin. Biotechnol..

[7] Bamforth SM, Singleton I (2005). Bioremediation of polycyclic aromatic hydrocarbons: Current knowledge and future directions. J. Chem. Technol. Biotechnol. Int. Res. Process Environ. Clean Technol..

[8] Das D (2015). Complete genome sequence analysis of *Pseudomonas aeruginosa* N002 reveals its genetic adaptation for crude oil degradation. Genomics.

[9] He C, Li Y, Huang C, Chen F, Ma Y (2018). Genome sequence and metabolic analysis of a fluoranthene-degrading strain *Pseudomonas aeruginosa* DN1. Front. Microbiol..

[10] Nie Y (2014). Diverse alkane hydroxylase genes in microorganisms and environments. Sci. Rep..

[11] Parte AC (2018). LPSN-list of prokaryotic names with standing in nomenclature (bacterio.net), 20 years on. Int. J. Syst. Evol. Microbiol..

[12] Gomila M, Peña A, Mulet M, Lalucat J, García-Valdés E (2015). Phylogenomics and systematics in Pseudomonas. Front. Microbiol..

[13] Xie F (2014). *Pseudomonas kunmingensis* sp. nov., an exopolysaccharide-producing bacterium isolated from a phosphate mine. Int. J. Syst. Evolut. Microbiol..

[14] Wolterink A, Jonker A, Kengen S, Stams A (2002). *Pseudomonas chloritidismutans* sp. nov., a non-denitrifying, chlorate-reducing bacterium. Int. J. Syst. Evolut. Microbiol..

[15] Coordinators NR (2016). Database resources of the national center for biotechnology information. Nucleic Acids Res..

[16] Mehboob F, Junca H, Schraa G, Stams AJM (2009). Growth of *Pseudomonas chloritidismutans* AW-1(T) on n-alkanes with chlorate as electron acceptor. Appl. Microbiol. Biotechnol..

[17] Mehboob F (2016). Genome and proteome analysis of *P. seudomonas* chloritidismutans AW-1 T that grows on n-decane with chlorate or oxygen as electron acceptor. Environ. Microbiol..

[18] Lagesen K (2007). RNAmmer: Consistent and rapid annotation of ribosomal RNA genes. Nucleic Acids Res..

[19] Yoon S-H (2017). Introducing EzBioCloud: A taxonomically united database of 16S rRNA gene sequences and whole-genome assemblies. Int. J. Syst. Evol. Microbiol..

[20] Hassen W (2018). *Pseudomonas rhizophila* S211, a new plant growth-promoting rhizobacterium with potential in pesticide-bioremediation. Front. Microbiol..

[21] Neifar M (2019). Genome analysis provides insights into crude oil degradation and biosurfactant production by extremely halotolerant *Halomonas desertis* G11 isolated from Chott El-Djerid salt-lake in Tunisian desert. Genomics.

[22] Wang S (2014). Coordination of swarming motility, biosurfactant synthesis, and biofilm matrix exopolysaccharide production in *Pseudomonas aeruginosa*. Appl. Environ. Microbiol..

[23] Paliwal V, Raju SC, Modak A, Phale PS, Purohit HJ (2014). Pseudomonas putida CSV86: A candidate genome for genetic bioaugmentation. PLoS ONE.

[24] Sopeña F (2014). Phenanthrene biodegradation by *Pseudomonas xanthomarina* isolated from an aged contaminated soil. Clean-Soil Air Water.

[25] Karimi B, Habibi M, Esvand M (2015). Biodegradation of naphthalene using *Pseudomonas aeruginosa* by up flow anoxic–aerobic continuous flow combined bioreactor. J. Environ. Health Sci. Eng..

[26] Kunihiro N, Haruki M, Takano K, Morikawa M, Kanaya S (2005). Isolation and characterization of *Rhodococcus* sp. strains TMP2 and T12 that degrade 2, 6, 10, 14-tetramethylpentadecane (pristane) at moderately low temperatures. J. Biotechnol..

[27] Bosch R, Moore ER, García-Valdés E, Pieper DH (1999). NahW, a novel, inducible salicylate hydroxylase involved in mineralization of naphthalene by *Pseudomonas stutzeri* AN10. J. Bacteriol..

[28] Dennis JJ, Zylstra GJ (2004). Complete sequence and genetic organization of pDTG1, the 83 kilobase naphthalene degradation plasmid from *Pseudomonas putida* strain NCIB 9816-4. J. Mol. Biol..

[29] Singleton DR, Ramirez LG, Aitken MD (2009). Characterization of a polycyclic aromatic hydrocarbon degradation gene cluster in a phenanthrene-degrading Acidovorax strain. Appl. Environ. Microbiol..

[30] Jain C, Rodriguez-R LM, Phillippy AM, Konstantinidis KT, Aluru S (2018). High throughput ANI analysis of 90K prokaryotic genomes reveals clear species boundaries. Nat. Commun..

[31] Konstantinidis KT, Tiedje JM (2005). Genomic insights that advance the species definition for prokaryotes. Proc. Natl. Acad. Sci. USA.

[32] Meier-Kolthoff JP, Auch AF, Klenk H-P, Göker M (2013). Genome sequence-based species delimitation with confidence intervals and improved distance functions. BMC Bioinform..

[33] Meier-Kolthoff JP, Klenk H-P, Göker M (2014). Taxonomic use of DNA G+C content and DNA–DNA hybridization in the genomic age. Int. J. Syst. Evol. Microbiol..

[34] Cladera AM, García-Valdés E, Lalucat J (2006). Genotype versus phenotype in the circumscription of bacterial species: The case of *Pseudomonas stutzeri* and *Pseudomonas chloritidismutans*. Arch. Microbiol..

[35] Lalucat J, Bennasar A, Bosch R, García-Valdés E, Palleroni NJ (2006). Biology of *Pseudomonas stutzeri*. Microbiol. Mol. Biol. Rev..

[36] Jain C, Rodriguez-R LM, Phillippy AM, Konstantinidis KT, Aluru S (2018). High throughput ANI analysis of 90K prokaryotic genomes reveals clear species boundaries. Nat. Commun..

[37] Kanehisa M, Sato Y, Morishima K (2016). BlastKOALA and GhostKOALA: KEGG tools for functional characterization of genome and metagenome sequences. J. Mol. Biol..

[38] Meier-Kolthoff JP, Auch AF, Klenk H-P, Göker M (2013). Genome sequence-based species delimitation with confidence intervals and improved distance functions. BMC Bioinform..

[39] Ridl J (2018). Complete genome sequence of Pseudomonas alcaliphila JAB1 (= DSM 26533), a versatile degrader of organic pollutants. Stand Genomic Sci..

[40] Tang, H. et al. Genome sequence of Pseudomonas putida strain B6-2, a superdegrader of polycyclic aromatic hydrocarbons and dioxin-like compounds. *J. Bacteriol.* **193**, 6789–6790 (2011). 10.1128/JB.06201-11 PMC3232905 22072645

[41] Kertesz, M. A., Kawasaki, A. & Stolz, A. Aerobic hydrocarbon-degrading alphaproteobacteria: Sphingomonadales. in *Taxonomy, Genomics and Ecophysiology of Hydrocarbon-Degrading Microbes* (ed. McGenity, T. J.) 105–124. 10.1007/978-3-030-14796-9_9 (2019).

[42] Lee Y, Lee Y, Jeon CO (2019). Biodegradation of naphthalene, BTEX, and aliphatic hydrocarbons by *Paraburkholderia aromaticivorans* BN5 isolated from petroleum-contaminated soil. Sci. Rep..

[43] Rochman FF (2017). Benzene and naphthalene degrading bacterial communities in an oil sands tailings pond. Front. Microbiol..

[44] Pathak A (2016). Comparative genomics and metabolic analysis reveals peculiar characteristics of *Rhodococcus opacus* strain M213 particularly for naphthalene degradation. PLoS ONE.

[45] Mahjoubi M (2019). The genome of *Alcaligenes aquatilis* strain BU33N: Insights into hydrocarbon degradation capacity. PLoS ONE.

[46] Zhang H (2018). dbCAN2: A meta server for automated carbohydrate-active enzyme annotation. Nucleic Acids Res..

[47] Marchler-Bauer A (2015). CDD: NCBI's conserved domain database. Nucleic Acids Res..

[48] Toribio J, Escalante AE, Soberón-Chávez G (2010). Rhamnolipids: Production in bacteria other than *Pseudomonas aeruginosa*. Eur. J. Lipid Sci. Technol..

[49] Santisi S (2015). Biodegradation of crude oil by individual bacterial strains and a mixed bacterial consortium. Braz. J. Microbiol..

[50] Katoh, K. & Standley, D. M. *Multiple Sequence Alignment Methods* 131–146 (Springer, 2014).

[51] Castresana J (2000). Selection of conserved blocks from multiple alignments for their use in phylogenetic analysis. Mol. Biol. Evol..

[52] Tamura K, Stecher G, Peterson D, Filipski A, Kumar S (2013). MEGA6: Molecular evolutionary genetics analysis version 6.0. Mol. Biol. Evolut..

[53] Stanton S, Meyer JJM, Van der Merwe CF (2013). An evaluation of the endophytic colonies present in pathogenic and non-pathogenic Vanguerieae using electron microscopy. S. Afr. J. Bot..

[54] Pinzon NM, Ju L-K (2009). Analysis of rhamnolipid biosurfactants by methylene blue complexation. Appl. Microbiol. Biotechnol..

[55] Youssef NH (2004). Comparison of methods to detect biosurfactant production by diverse microorganisms. J. Microbiol. Methods.

[56] Mathieu, D., Nony, J. & Phan-Tan-Luu, R. *Nemrod-W Software* (LPRAI, 2000).

[57] Ghorbel RE, Kamoun A, Neifar M, Chaabouni SE (2010). Optimization of new flour improver mixing formula by surface response methodology. J. Food Process Eng.

[58] Rahman PK, Pasirayi G, Auger V, Ali Z (2010). Production of rhamnolipid biosurfactants by *Pseudomonas aeruginosa* DS10-129 in a microfluidic bioreactor. Biotechnol. Appl. Biochem..

[59] Genovese M (2014). Effective bioremediation strategy for rapid in situ cleanup of anoxic marine sediments in mesocosm oil spill simulation. Front. Microbiol..

[60] Hassanshahian M, Emtiazi G, Caruso G, Cappello S (2014). Bioremediation (bioaugmentation/biostimulation) trials of oil polluted seawater: A mesocosm simulation study. Mar. Environ. Res..

[61] Bankevich A (2012). SPAdes: A new genome assembly algorithm and its applications to single-cell sequencing. J. Comput. Biol..

[62] Seemann T (2014). Prokka: Rapid prokaryotic genome annotation. Bioinformatics.

[63] Aziz RK (2008). The RAST Server: Rapid annotations using subsystems technology. BMC Genom..

[64] Kanehisa M, Sato Y, Furumichi M, Morishima K, Tanabe M (2019). New approach for understanding genome variations in KEGG. Nucleic Acids Res..

[65] Kanehisa M, Goto S (2000). KEGG: Kyoto encyclopedia of genes and genomes. Nucleic Acids Res..

[66] Huerta-Cepas, J. et al. Fast genome-wide functional annotation through orthology assignment by eggNOG-mapper. *Mol. Biol. Evol.* **34**, 2115–2122 (2017). 10.1093/molbev/msx148 PMC5850834 28460117

[67] Emms DM, Kelly S (2015). OrthoFinder: Solving fundamental biases in whole genome comparisons dramatically improves orthogroup inference accuracy. Genom. Biol..

[68] Capella-Gutiérrez S, Silla-Martínez JM, Gabaldón T (2009). trimAl: A tool for automated alignment trimming in large-scale phylogenetic analyses. Bioinformatics.

[69] Lee I, Ouk Kim Y, Park S-C, Chun J (2016). OrthoANI: An improved algorithm and software for calculating average nucleotide identity. Int. J. Syst. Evol. Microbiol..

[70] Veltri D, Wight MM, Crouch JA (2016). SimpleSynteny: A web-based tool for visualization of microsynteny across multiple species. Nucleic Acids Res..

[71] Metsalu T, Vilo J (2015). ClustVis: A web tool for visualizing clustering of multivariate data using principal component analysis and heatmap. Nucleic Acids Res..

