# Supplementary material for: Genomic characterization of a polyvalent hydrocarbonoclastic bacterium Pseudomonas sp. strain BUN14
Source: Sci Rep. 2021 Apr 14;11:8124. doi: 10.1038/s41598-021-87487-2 (PMC8046798; doi:10.1038/s41598-021-87487-2)
Supplement: Supplementary file 1 — Supplementary Information. [file 41598_2021_87487_MOESM1_ESM.doc]

## Supplementary Information

# Genomic characterization of a polyvalent hydrocarbonoclastic bacterium *Pseudomonas* sp. strain BUN14

Mouna Mahjoubi^1^, Habibu Aliyu^2^, Mohamed Neifar^1^, Simone Cappello^3^, Habib Chouchane^1^, Yasmine Souissi^1^, Ahmed Salaheddine Masmoudi^1^, Don A. Cowan^4^, Ameur Cherif ^1*^

*^1^Univ. Manouba, ISBST, BVBGR-LR11ES31, Biotechpole SidiThabet, 2020, Ariana, Tunisia.*

*^2^Institute of Process Engineering in Life Science 2: Technical Biology, Karlsruhe Institute of Technology, Karlsruhe, Germany.*

*^3^Istituto per le Risorse Biologiche e le Biotecnologie Marine (IRBIM) – CNR of Messina. Sp. San Raineri, 86 - 98122 Messina, Italy.*

*^4^Centre for Microbial Ecology and Genomics, University of Pretoria, Pretoria 0002, South Africa.*

** Correspondence: A. Cherif, Tel./Fax: +216 70 527 882, E-mail:* [***cherif.ameur@gmail.com***](mailto:cherif.ameur@gmail.com)


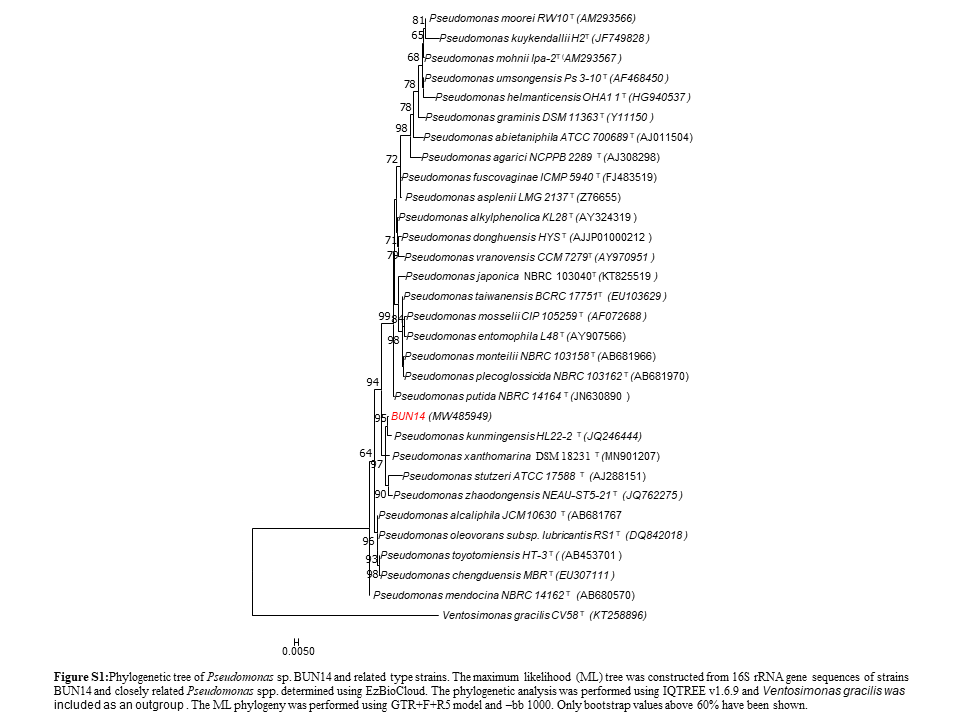


**Figure S1:**Phylogenetic tree of *Pseudomonas* sp. BUN14 and related type strains. The maximum likelihood (ML) tree was constructed from 16S rRNA gene sequences of strains BUN14 and closely related *Pseudomonas* spp. determined using EzBioCloud. The phylogenetic analysis was performed using IQTREE v1.6.9 and *Ventosimonas gracilis* was included as an outgroup. The ML phylogeny was performed using GTR+F+R5 model and –bb 1000. Only bootstrap values above 60% have been shown.


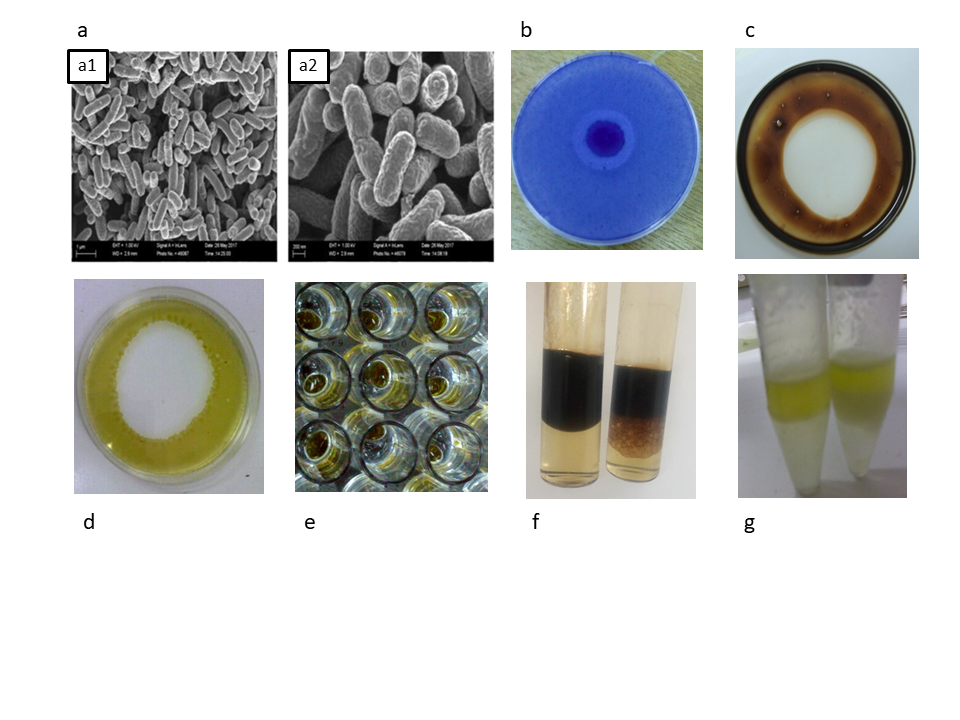


**Figure S2:** Characterization of *Pseudomonas* BUN14:   a)  Scanning electron micrographs of strain BUN14 grown on TSA medium (at 30 ±1°C for 48h, a1 bar = 1µm and a2 bar = 200nm), Biosurfactant production by BUN14: b) CTAB test, c) ODA with crude oil, d) ODA with vegetable oil, e) drop collapse test , f) E24% with crude oil and g ) E24% with vegetable oil.


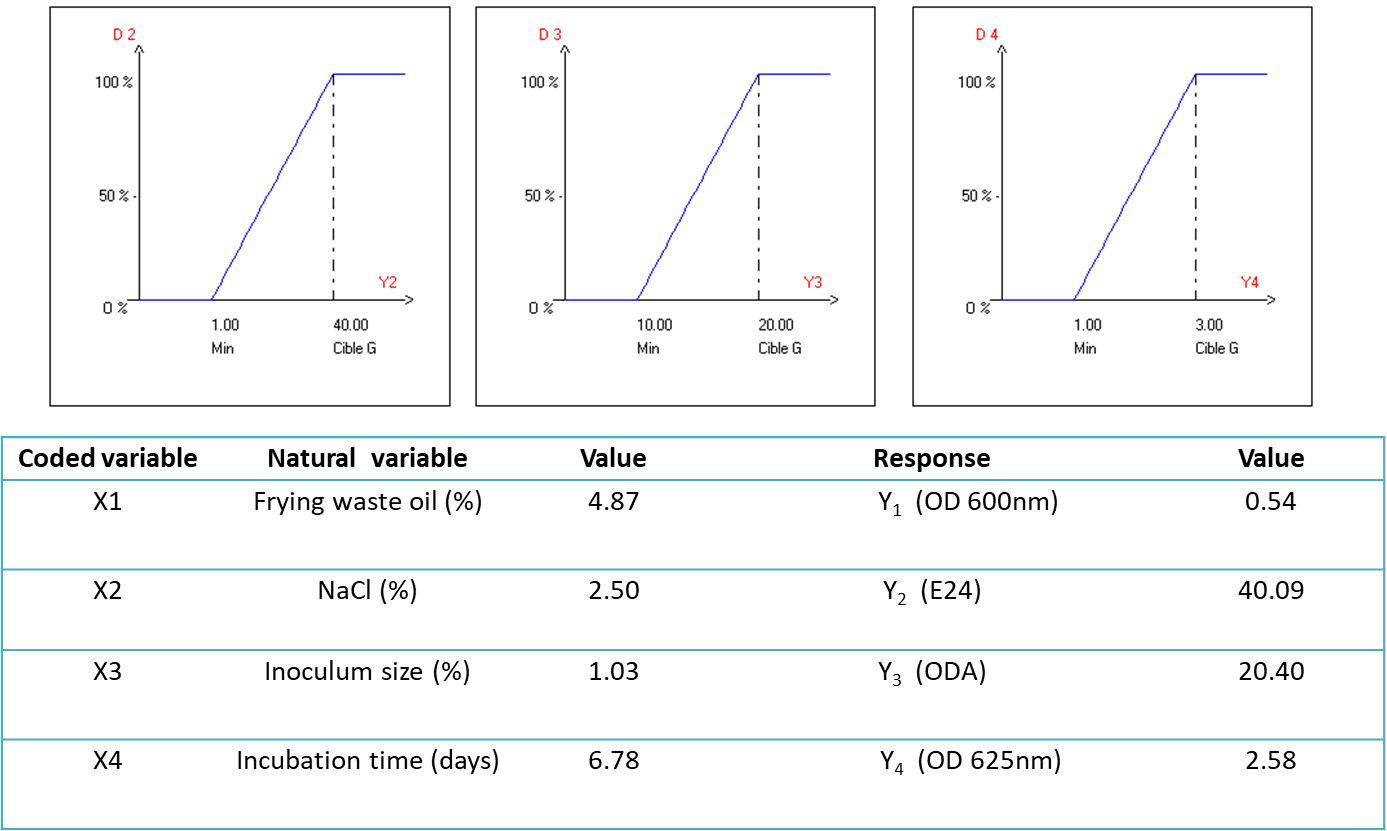


**Figure S3:** Optimized operational conditions for maximum biosurfactant production determined on the basis of desirability functions


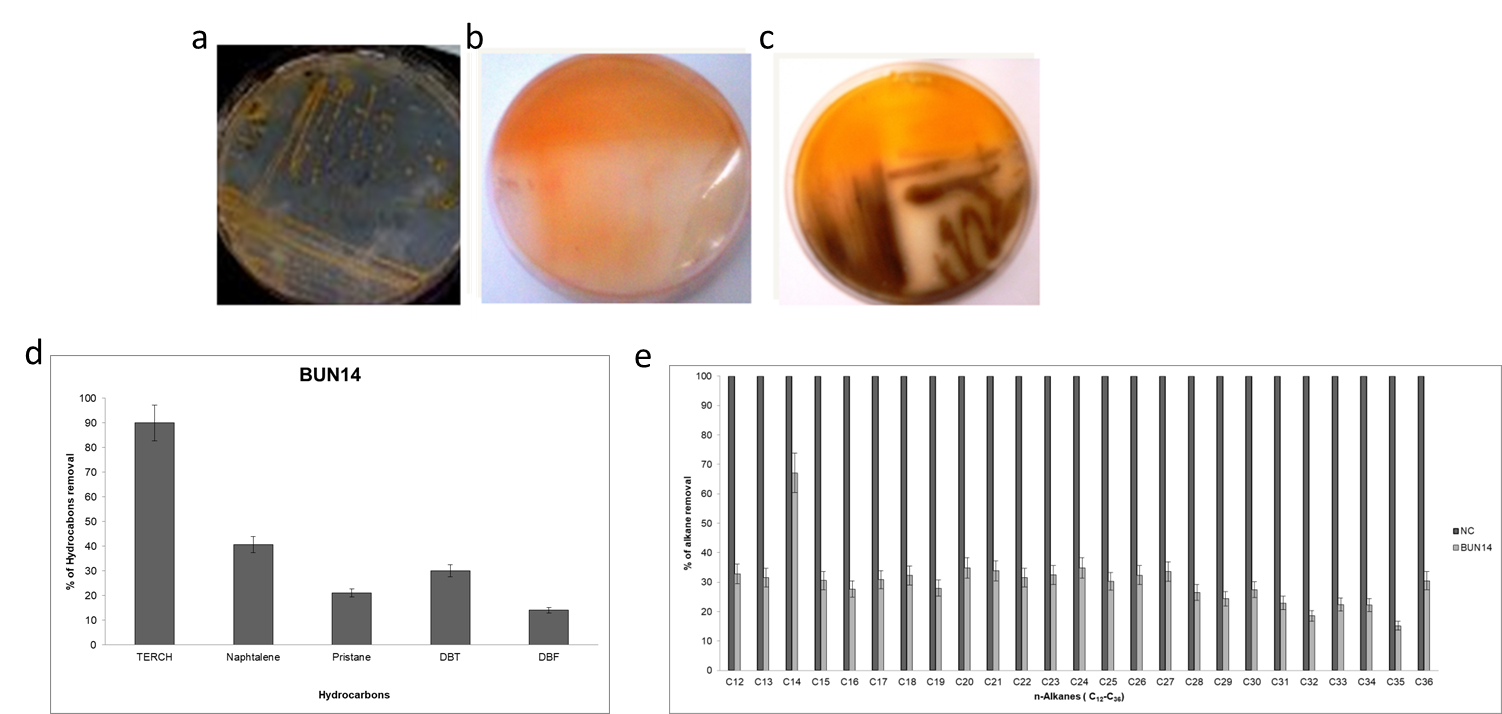


**Figure S4:** Growth of *Pseudomonas* BUN14 on ONR7a medium supplemented with different hydrocarbons (a) crude oil, (b) dibenzothiophene, (c) dibenzofuran ,relative percentage of TERHc, Naphtalene , pristame DBT , DBF ( d) and alkane (e) by BUN14


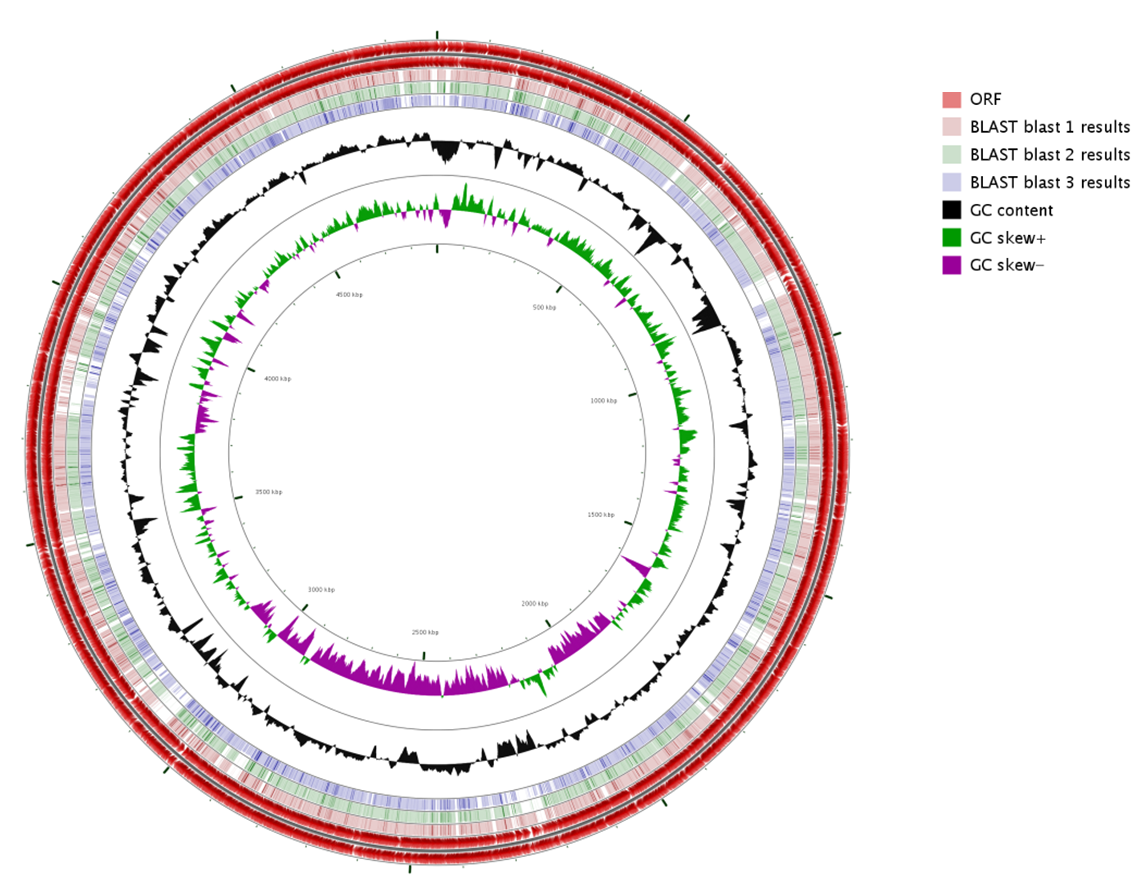


**Figure S5:** **Figure S5**. BLAST comparison of draft genome of *Pseudomonas.* BUN14 against three *Pseudomonas* species. The innermost rings depict GC content (Black) and GC Skew (purple/green) followed by concentric rings of query sequences colored according to BLAST identity. The outermost rings depict genomes of the following microbes of *P. kunmingensis* DSM 25974 (Pink), *P. kunmingensis* CCUG 36651 (Green) and *P. chloritidismutans* AW-1 *P. (*Blue). The circular map was constructed using GC View beta version (http://stothard.afns.ualberta.ca/cgview_server/)


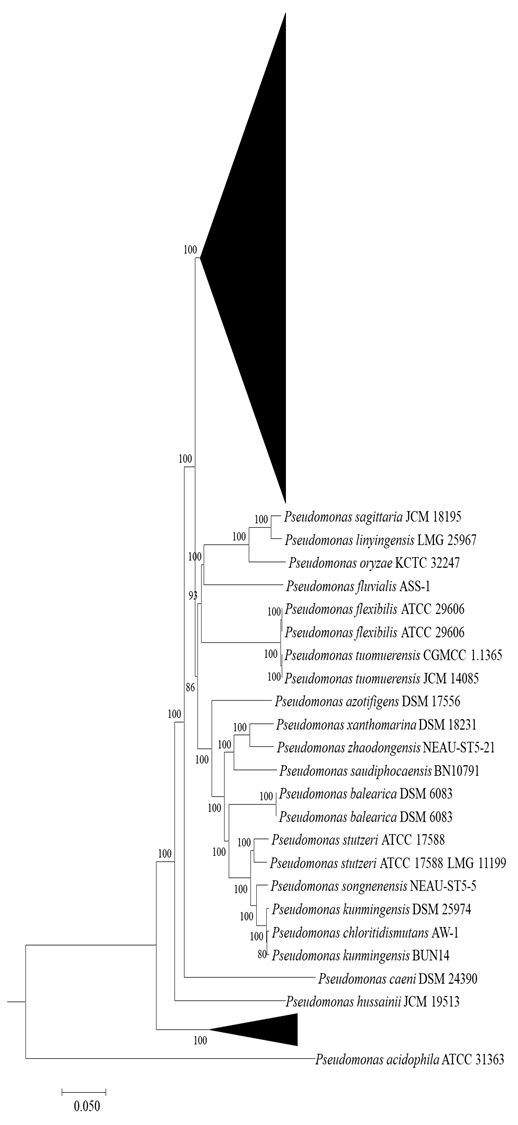


**Figure S6:** Phylogenomic guide tree constructed based on 189 shared single copy proteins from 294 *Pseudomonas* type strain obtained from the NCBI database. The tree was rooted at midpoint and only the branch including *Pseudomonas* strain BUN14 and closely related strains relevant to this work have has been shown. The close triangles contain other *Pseudomonas* species that are not included in the current work


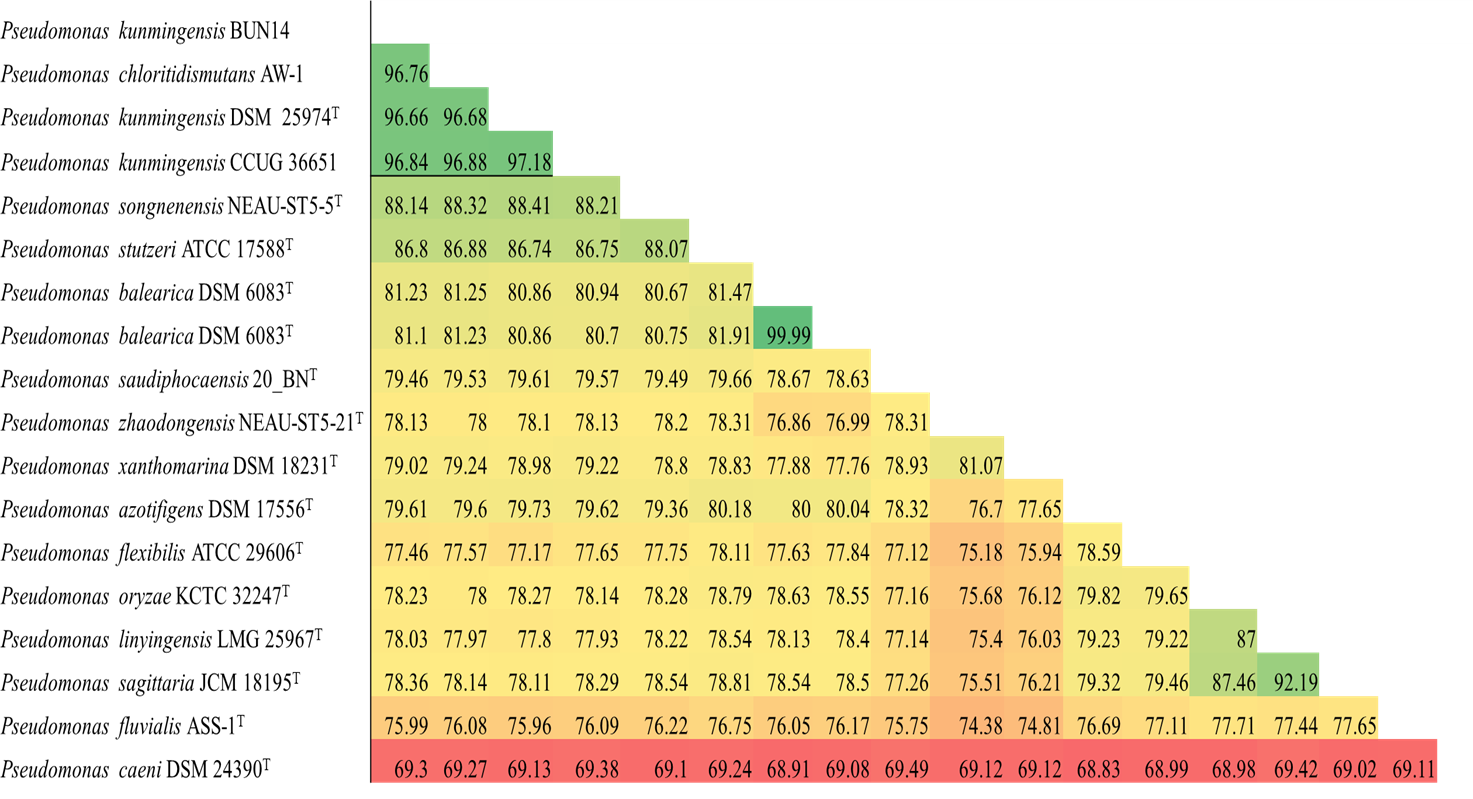


**Figure S7:** Comparison of average nucleotide identities (%) between seventeen *Pseudomonas* strains, including strain BUN14 and its closest relatives.

**Figure S8**: *In silico* DDH showing the genomic relatedness of *Pseudomonas* strain BUN14 and closest type strains based determined using Genome-to-Genome Distance calculator

**Figure S9**. Functional annotation of strain BUN14 proteome using KEGG database. The bar chart shows the distribution of proteins assigned to various metabolic pathways. Excel with a summary of features from KEGG annotation was used to elaborate the figure.


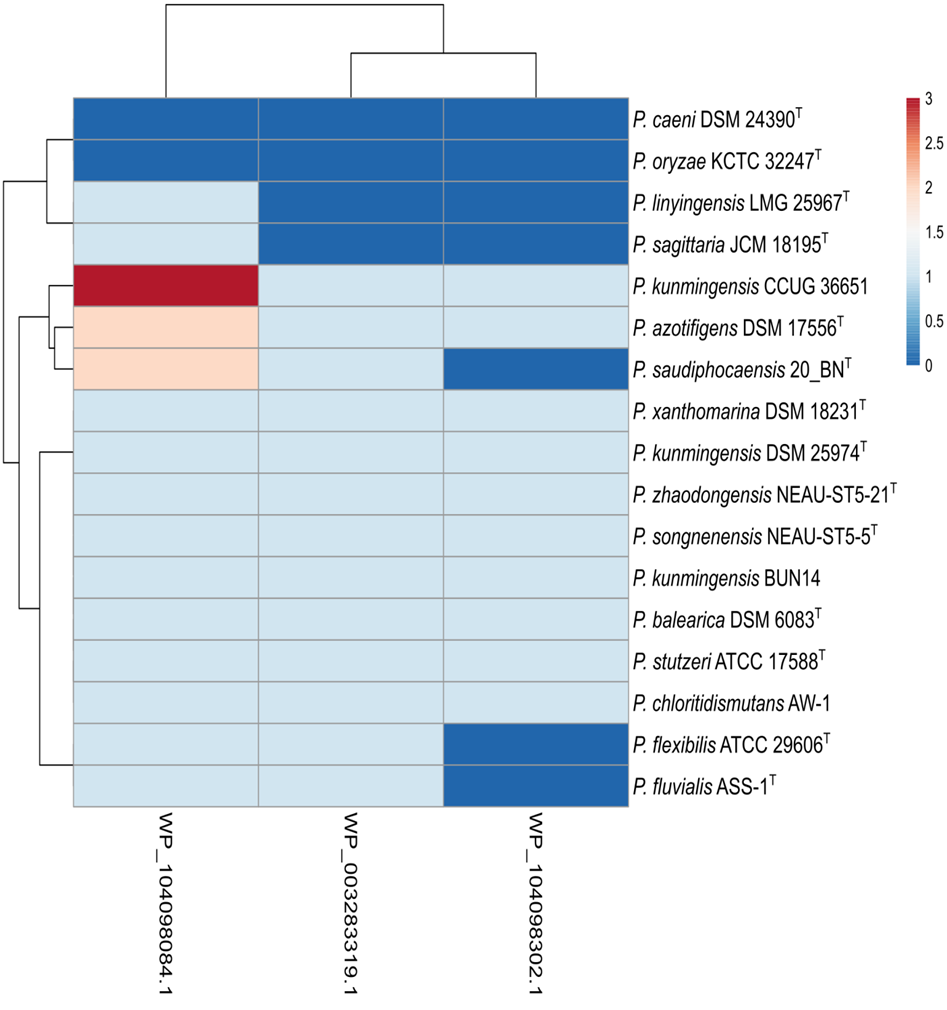


**Figure S10.** Heat map showing the distribution of the predicted proteins linked to alkane degradation pathway in strain BUN14 and orthologous of the proteins among closely related species. The heat map was generated based on the counts of orthologous proteins determined using Orthofinder. Heat map was produced using Clustvis version 1 (<https://biit.cs.ut.ee/clustvis/>)

**Table S1**. Number of genes associated with general EggNOG functional categories

| **COG class and description** | **count** | **% count** |
| --- | --- | --- |
| CELLULAR PROCESSES AND SIGNALING |  |  |
| [D] Cell cycle control, cell division, chromosome partitioning | 35 | 0.87 |
| [M] Cell wall/membrane/envelope biogenesis | 220 | 5.47 |
| [N] Cell motility | 74 | 1.84 |
| [O] Post-translational modification, protein turnover, and chaperones | 171 | 4.25 |
| [T] Signal transduction mechanisms | 254 | 6.31 |
| [U] Intracellular trafficking, secretion, and vesicular transport | 100 | 2.48 |
| [V] Defense mechanisms | 67 | 1.66 |
|  | **921** | **22.88** |
| INFORMATION STORAGE AND PROCESSING |  |  |
| [A] RNA processing and modification | 1 | 0.02 |
| [B] Chromatin structure and dynamics | 1 | 0.02 |
| [J] Translation, ribosomal structure, and biogenesis | 179 | 4.45 |
| [K] Transcription | 266 | 6.61 |
| [L] Replication, recombination, and repair | 221 | 5.49 |
|  | **668** | **16.60** |
| METABOLISM |  |  |
| [C] Energy production and conversion | 270 | 6.71 |
| [E] Amino acid transport and metabolism | 274 | 6.81 |
| [F] Nucleotide transport and metabolism | 97 | 2.41 |
| [G] Carbohydrate transport and metabolism | 166 | 4.12 |
| [H] Coenzyme transport and metabolism | 135 | 3.35 |
| [I] Lipid transport and metabolism | 134 | 3.33 |
| [P] Inorganic ion transport and metabolism | 260 | 6.46 |
| [Q] Secondary metabolites biosynthesis, transport, and catabolism | 89 | 2.21 |
|  | **1425** | **35.40** |
| POORLY CHARACTERIZED |  |  |
| [S] Function unknown | **1011** | **25.12** |

**Table S2**. Key enzymes associated with aromatic hydrocarbon degradation encoded in the *Pseudomonas* BUN14 genome

| BUN14_query | Protein ID | KO | Definition | Definition | Degradation |
| --- | --- | --- | --- | --- | --- |
| HPCCOKHE_01208 | WP_014820710.1 | K00363 | nirD; nitrite reductase (NADH) small subunit [EC:1.7.1.15] (HPCCOKHE_03657) * | nirD | Naphthalene |
| HPCCOKHE_02074 | WP_104097840.1 |  | HTH-type transcriptional activator RhaS |  |  |
| HPCCOKHE_02075 | WP_041013671.1 | K05549 | benA-xylX; benzoate/toluate 1,2-dioxygenase subunit alpha [EC:1.14.12.10 1.14.12.-] (HPCCOKHE_03656) * | benA-xylX | Benzoate/naphthalene |
| HPCCOKHE_02077 | WP_041013672.1 | K05550 | benB-xylY; benzoate/toluate 1,2-dioxygenase subunit beta [EC:1.14.12.10 1.14.12.-] | benB-xylY | Benzoate |
| HPCCOKHE_02078 | WP_058065630.1 | K05784 | benC-xylZ; benzoate/toluate 1,2-dioxygenase reductase component [EC:1.18.1.-] | benC-xylZ | Benzoate |
| HPCCOKHE_02079 | WP_104097841.1 | K05783 | benD-xylL; dihydroxycyclohexadiene carboxylate dehydrogenase [EC:1.3.1.25 1.3.1.-] | benD-xylL | Benzoate |
| HPCCOKHE_02080 | WP_104097842.1 | K05548 | benK; MFS transporter, AAHS family, benzoate transport protein | benK | Benzoate |
| HPCCOKHE_02081 | WP_104097843.1 | K01856 | catB; muconate cycloisomerase [EC:5.5.1.1] | catB | Benzoate |
| HPCCOKHE_02082 | WP_023445688.1 | K03464 | catC; muconolactone D-isomerase [EC:5.3.3.4] | catC | Benzoate |
| HPCCOKHE_02083 | WP_104097844.1 | K03381 | catA; catechol 1,2-dioxygenase [EC:1.13.11.1] | catA | Benzoate |
| HPCCOKHE_03542 | WP_023443939.1 | K01055 | pcaD; 3-oxoadipate enol-lactonase [EC:3.1.1.24] | pcaD | Benzoate |
| HPCCOKHE_03621 | WP_003292104.1 |  | Haem_degrading superfamily |  |  |
| HPCCOKHE_03622 | WP_009397185.1 | K01821 | praC; 4-oxalocrotonate tautomerase [EC:5.3.2.6] | praC | Benzoate/dioxin |
| HPCCOKHE_03623 | WP_009397184.1 | K01617 | dmpH; 2-oxo-3-hexenedioate decarboxylase [EC:4.1.1.77] | dmpH | Benzoate/dioxin |
| HPCCOKHE_03624 | WP_104098552.1 | K18365 | bphI; 4-hydroxy-2-oxovalerate/4-hydroxy-2-oxohexanoate aldolase [EC:4.1.3.39 4.1.3.43] | bphI | Benzoate/dioxin |
| HPCCOKHE_03625 | WP_003292099.1 | K18366 | bphJ; acetaldehyde/propanal dehydrogenase [EC:1.2.1.10 1.2.1.87] | bphJ | Benzoate/dioxin |
| HPCCOKHE_03626 | WP_003292096.1 | K18364 | bphH; 2-oxopent-4-enoate/cis-2-oxohex-4-enoate hydratase [EC:4.2.1.80 4.2.1.132] | bphH | Benzoate/dioxin |
| HPCCOKHE_03627 | WP_003292095.1 | K10216 | dmpD; 2-hydroxymuconate-semialdehyde hydrolase [EC:3.7.1.9] | dmpD | Benzoate |
| HPCCOKHE_03628 | WP_104098553.1 | K10217 | dmpC; aminomuconate-semialdehyde/2-hydroxymuconate-6-semialdehyde dehydrogenase [EC:1.2.1.32 1.2.1.85] | dmpC | Benzoate |
| HPCCOKHE_03629 | WP_003292092.1 | K00446 | dmpB; catechol 2,3-dioxygenase [EC:1.13.11.2] | dmpB | Benzoate |
| HPCCOKHE_03630 | WP_003292091.1 |  | fer2 superfamily |  | Benzoate |
| HPCCOKHE_03631 | WP_014819623.1 | K00480 | E1.14.13.1; salicylate hydroxylase [EC:1.14.13.1] | E1.14.13.1 | Dioxin |
| HPCCOKHE_03635 | WP_104098554.1 | K00480 | E1.14.13.1; salicylate hydroxylase [EC:1.14.13.1] | E1.14.13.1 | Dioxin |
| HPCCOKHE_03650 | WP_003292062.1 | K14584 | nahD; 2-hydroxychromene-2-carboxylate isomerase [EC:5.99.1.4] | nahD | Naphthalene |
| HPCCOKHE_03651 | WP_003292061.1 | K14585 | nahE; trans-o-hydroxybenzylidenepyruvate hydratase-aldolase [EC:4.1.2.45] | nahE | Naphthalene |
| HPCCOKHE_03652 | WP_104098556.1 | K14583 | nahC; 1,2-dihydroxynaphthalene dioxygenase [EC:1.13.11.56] | nahC | Naphthalene |
| HPCCOKHE_03653 | WP_104098557.1 | K00152 | nahF; salicylaldehyde dehydrogenase [EC:1.2.1.65] | nahF | Naphthalene |
| HPCCOKHE_03654 | WP_104098558.1 | K14582 | nahB; cis-1,2-dihydro-1,2-dihydroxynaphthalene/dibenzothiophene dihydrodiol dehydrogenase [EC:1.3.1.29 1.3.1.60] | nahB | Naphthalene |
| HPCCOKHE_03655 | WP_104098559.1 | K14580 | nahAd; naphthalene 1,2-dioxygenase subunit beta [EC:1.14.12.12 1.14.12.23 1.14.12.24] | nahAd | Naphthalene |
| HPCCOKHE_03656 | WP_104098560.1 | K14579 | nahAc; naphthalene 1,2-dioxygenase subunit alpha [EC:1.14.12.12 1.14.12.23 1.14.12.24] | nahAc | Naphthalene |
| HPCCOKHE_03657 | WP_009399901.1 | K14578 | nahAb; naphthalene 1,2-dioxygenase ferredoxin component | nahAb | Naphthalene |
| HPCCOKHE_03658 | WP_009399900.1 | K14581 | nahAa; naphthalene 1,2-dioxygenase ferredoxin reductase component [EC:1.18.1.7] | nahAa | Naphthalene |
| HPCCOKHE_03925 | WP_104098726.1 |  | Haem_degrading superfamily |  |  |
| HPCCOKHE_03926 | WP_104098727.1 | K01821 | praC; 4-oxalocrotonate tautomerase [EC:5.3.2.6] | praC | Benzoate/dioxin |
| HPCCOKHE_03927 | WP_104098728.1 | K01617 | dmpH; 2-oxo-3-hexenedioate decarboxylase [EC:4.1.1.77] | dmpH | Benzoate/dioxin |
| HPCCOKHE_03928 | WP_004574815.1 | K01666 | mhpE; 4-hydroxy 2-oxovalerate aldolase [EC:4.1.3.39] | mhpE | Benzoate/dioxin |
| HPCCOKHE_03929 | WP_104098729.1 | K04073 | mhpF; acetaldehyde dehydrogenase [EC:1.2.1.10] | mhpF | Benzoate/dioxin |
| HPCCOKHE_03930 | WP_104098730.1 | K18364 | bphH; 2-oxopent-4-enoate/cis-2-oxohex-4-enoate hydratase [EC:4.2.1.80 4.2.1.132] | bphH | Benzoate/dioxin |
| HPCCOKHE_03931 | WP_104098731.1 | K10216 | dmpD; 2-hydroxymuconate-semialdehyde hydrolase [EC:3.7.1.9] | dmpD | Benzoate |
| HPCCOKHE_03932 | WP_104098732.1 | K10217 | dmpC; aminomuconate-semialdehyde/2-hydroxymuconate-6-semialdehyde dehydrogenase [EC:1.2.1.32 1.2.1.85] | dmpC | Benzoate |
| HPCCOKHE_03933 | WP_104098733.1 | K00446 | dmpB; catechol 2,3-dioxygenase [EC:1.13.11.2] | dmpB | Benzoate |
| HPCCOKHE_03934 | WP_096425583.1 |  | fer2 superfamily |  |  |
| HPCCOKHE_03935 | WP_104098734.1 | K16246 | dmpP; phenol/toluene 2-monooxygenase (NADH) P5/A5 [EC:1.14.13.244 1.14.13.243] | dmpP | Benzoate |
| HPCCOKHE_03936 | WP_033045113.1 | K16245 | dmpO; phenol/toluene 2-monooxygenase (NADH) P4/A4 [EC:1.14.13.244 1.14.13.243] | dmpO | Benzoate |
| HPCCOKHE_03937 | WP_060489526.1 | K16242 | dmpN; phenol/toluene 2-monooxygenase (NADH) P3/A3 [EC:1.14.13.244 1.14.13.243] | dmpN | Benzoate |
| HPCCOKHE_03938 | WP_060489530.1 | K16244 | dmpM; phenol/toluene 2-monooxygenase (NADH) P2/A2 [EC:1.14.13.244 1.14.13.243] | dmpM | Benzoate |
| HPCCOKHE_03939 | WP_023086561.1 | K16243 | dmpL; phenol/toluene 2-monooxygenase (NADH) P1/A1 [EC:1.14.13.244 1.14.13.243] | dmpL | Benzoate |
| HPCCOKHE_03940 | WP_062381239.1 | K16249 | dmpK; phenol/toluene 2-monooxygenase (NADH) P0/A0 | dmpK | Benzoate |
| HPCCOKHE_04505 | WP_104099050.1 | K09023 | rutD; aminoacrylate hydrolase [EC:3.5.1.-] (HPCCOKHE_03542) * | rutD | Benzoate |

**Table S3**. Putative key rhamnolipid biosynthesis enzymes encoded in the *Pseudomonas* strain BUN14 genome and their orthologous relationships with proteins from closely related strains

| Protein ID | WP_104098192.1 | WP_104098902.1 | WP_042926909.1 | WP_003300529.1 |
| --- | --- | --- | --- | --- |
| Definition | alpha/beta hydrolase | glycosyltransferase family 1 protein | glycosyltransferase family 2 protein | glycosyltransferase |
| *Pseudomonas kunmingensis* DSM 25974^T^ | 1 | 1 | 1 | 1 |
| *Pseudomonas chloritidismutans* AW-1 | 1 | 1 | 0 | 1 |
| *Pseudomonas kunmingensis* CCUG 36651 | 1 | 1 | 1 | 1 |
| *Pseudomonas kunmingensis* BUN14 | 1 | 1 | 1 | 1 |
| *Pseudomonas songnenensis* NEAU-ST5-5 ^T^ | 1 | 1 | 1 | 1 |
| *Pseudomonas stutzeri* ATCC 17588 ^T^ | 1 | 1 | 1 | 1 |
| *Pseudomonas balearica* DSM 6083 ^T^ | 1 | 0 | 1 | 1 |
| *Pseudomonas saudiphocaensis* 20_BN ^T^ | 0 | 1 | 1 | 1 |
| *Pseudomonas zhaodongensis* NEAU-ST5-21 ^T^ | 0 | 1 | 1 | 1 |
| *Pseudomonas xanthomarina* DSM 18231 ^T^ | 0 | 1 | 1 | 1 |
| *Pseudomonas azotifigens* DSM 17556 ^T^ | 1 | 0 | 1 | 1 |
| *Pseudomonas flexibilis* ATCC 29606 ^T^ | 0 | 0 | 0 | 0 |
| *Pseudomonas oryzae* KCTC 32247 ^T^ | 0 | 0 | 0 | 0 |
| *Pseudomonas linyingensis* LMG 25967 ^T^ | 0 | 0 | 0 | 0 |
| *Pseudomonas sagittaria* JCM 18195 ^T^ | 0 | 0 | 0 | 0 |
| *Pseudomonas fluvialis* ASS-1 ^T^ | 0 | 0 | 0 | 0 |
| *Pseudomonas caeni* DSM 24390 ^T^ | 0 | 0 | 0 | 0 |

***Table S4:*** *Characteristics of the central composite experimental design used for the optimization of rhamnolipid production by BUN14*

| **Variable** | **Factor** | **Unit** | **Center** | **Step of variation** |
| --- | --- | --- | --- | --- |
| X1 | Frying waste oil | % | 3 | 2 |
| X2 | NaCl | % | 3.5 | 3.5 |
| X3 | Inoculum size | % | 2 | 1 |
| X4 | Incubation time | days | 4 | 3 |

**Table S5:** Experimental conditions of the central composite design and the corresponding responses

| **N° Exp** | **Frying waste oil** | **NaCl** | **Inoculum size** | **Incubation time** | **OD 600nm** | **E24** | **ODA** | **OD 625nm** |
| --- | --- | --- | --- | --- | --- | --- | --- | --- |
|  | (%) | (%) | (%) | (j) | - | (%) | (cm^2^) | - |
| 1 | 1.00 | 0.00 | 1.00 | 1.00 | 1.65 | 29.00 | 19.02 | 0.82 |
| 2 | 5.00 | 0.00 | 1.00 | 1.00 | 2.51 | 40.00 | 17.30 | 1.49 |
| 3 | 1.00 | 7.00 | 1.00 | 1.00 | 0.78 | 10.00 | 6.90 | 0.19 |
| 4 | 5.00 | 7.00 | 1.00 | 1.00 | 1.39 | 30.00 | 9.61 | 0.91 |
| 5 | 1.00 | 0.00 | 3.00 | 1.00 | 0.11 | 35.00 | 12.20 | 0.89 |
| 6 | 5.00 | 0.00 | 3.00 | 1.00 | 0.78 | 16.00 | 8.14 | 0.59 |
| 7 | 1.00 | 7.00 | 3.00 | 1.00 | 0.40 | 19.00 | 14.06 | 0.68 |
| 8 | 5.00 | 7.00 | 3.00 | 1.00 | 1.59 | 16.00 | 15.89 | 0.74 |
| 9 | 1.00 | 0.00 | 1.00 | 7.00 | 2.44 | 26.00 | 16.05 | 2.46 |
| 10 | 5.00 | 0.00 | 1.00 | 7.00 | 2.23 | 40.00 | 19.62 | 2.51 |
| 11 | 1.00 | 7.00 | 1.00 | 7.00 | 0.39 | 18.00 | 3.50 | 2.12 |
| 12 | 5.00 | 7.00 | 1.00 | 7.00 | 0.11 | 33.00 | 15.89 | 3.10 |
| 13 | 1.00 | 0.00 | 3.00 | 7.00 | 1.12 | 22.00 | 10.77 | 1.00 |
| 14 | 5.00 | 0.00 | 3.00 | 7.00 | 2.50 | 18.00 | 14.06 | 0.38 |
| 15 | 1.00 | 7.00 | 3.00 | 7.00 | 0.81 | 26.00 | 10.84 | 1.20 |
| 16 | 5.00 | 7.00 | 3.00 | 7.00 | 0.10 | 24.00 | 16.61 | 1.06 |
| 17 | 1.00 | 3.50 | 2.00 | 4.00 | 1.09 | 22.00 | 15.89 | 0.68 |
| 18 | 5.00 | 3.50 | 2.00 | 4.00 | 0.87 | 36.00 | 18.08 | 0.97 |
| 19 | 3.00 | 0.00 | 2.00 | 4.00 | 3.33 | 32.00 | 19.62 | 0.63 |
| 20 | 3.00 | 7.00 | 2.00 | 4.00 | 2.33 | 24.00 | 14.51 | 0.59 |
| 21 | 3.00 | 3.50 | 1.00 | 4.00 | 1.11 | 40.00 | 19.61 | 1.55 |
| 22 | 3.00 | 3.50 | 3.00 | 4.00 | 1.36 | 30.00 | 21.22 | 0.63 |
| 23 | 3.00 | 3.50 | 2.00 | 1.00 | 0.86 | 23.00 | 13.19 | 0.10 |
| 24 | 3.00 | 3.50 | 2.00 | 7.00 | 1.00 | 20.00 | 18.08 | 0.72 |
| 25 | 3.00 | 3.50 | 2.00 | 4.00 | 1.65 | 36.00 | 17.34 | 0.68 |
| 26 | 3.00 | 3.50 | 2.00 | 4.00 | 1.04 | 30.00 | 19.62 | 0.62 |
| 27 | 3.00 | 3.50 | 2.00 | 4.00 | 0.96 | 31.00 | 18.08 | 0.82 |
| 28 | 3.00 | 3.50 | 2.00 | 4.00 | 1.54 | 30.00 | 17.34 | 0.79 |
| 29 | 3.00 | 3.50 | 2.00 | 4.00 | 0.98 | 29.00 | 15.89 | 0.71 |

**Table S6:** Analysis of variance of the central composite design

| **Response** | **Source of variation** | **Sum of squares** | **df** | **Mean square** | **Ratio** | **Significance** |
| --- | --- | --- | --- | --- | --- | --- |
| **Y1: OD 600nm** | Regression | 15.8292 | 14 | 1.1307 | 6.7015 | ******* |
|  | Residuals | 2.3620 | 14 | 0.1687 |  |  |
|  | Lac of fit | 1.9181 | 10 | 0.1918 | 1.7283 | NS |
|  | Error | 0.4439 | 4 | 0.1110 |  |  |
|  | Total | 18.1912 | 28 |  |  |  |
| **Y2: E24 (%)** | Regression | 1581.19 | 14 | 112.942 | 9.6019 | ******* |
|  | Residuals | 164.674 | 14 | 11.7624 |  |  |
|  | Lac of fit | 133.874 | 10 | 13.3873 | 1.7386 | NS |
|  | Error | 30.8000 | 4 | 7.70000 |  |  |
|  | Total | 1745.86 | 28 |  |  |  |
| **Y3: ODA (cm^2^)** | Regression | 475.8364 | 14 | 33.9883 | 2.6478 | ******* |
|  | Residuals | 37.6221 | 14 | 2.6873 |  |  |
|  | Lac of fit | 30.2665 | 10 | 3.0267 | 1.6459 | NS |
|  | Error | 7.3555 | 4 | 1.8389 |  |  |
|  | Total | 513.4585 | 28 |  |  |  |
| **Y4: OD 625nm** | Regression | 13.6604 | 14 | 0.9757 | 52.4470 | ******* |
|  | Residuals | 0.2605 | 14 | 0.0186 |  |  |
|  | Lac of fit | 0.2339 | 10 | 0.0234 | 3.5285 | NS |
|  | Error | 0.0265 | 4 | 0.0066 |  |  |
|  | Total | 13.9208 | 28 |  |  |  |

***∗∗∗* Significant at the level 99.9% N.S.: nonsignificant**
